# The viral nucleocapsid protein and the human RNA-binding protein Mex3A promote translation of the *Andes orthohantavirus* small mRNA

**DOI:** 10.1371/journal.ppat.1009931

**Published:** 2021-09-21

**Authors:** Jorge Vera-Otarola, Estefania Castillo-Vargas, Jenniffer Angulo, Francisco M. Barriga, Eduard Batlle, Marcelo Lopez-Lastra

**Affiliations:** 1 Laboratorio de Virología Molecular, Instituto Milenio de Inmunología e Inmunoterapia, Departamento de Enfermedades Infecciosas e Inmunología Pediátrica, Centro de Investigaciones Médicas, Escuela de Medicina, Pontificia Universidad Católica de Chile, Santiago, Chile; 2 Unidad de Virología Aplicada, Dirección de Investigación y Doctorados de la Escuela de Medicina, Pontificia Universidad Católica de Chile, Santiago, Chile; 3 Facultad de Odontología, Universidad Finis Terrae, Santiago, Chile; 4 Institute for Research in Biomedicine (IRB Barcelona). The Barcelona Institute of Science and Technology. Barcelona, Spain; 5 ICREA, Barcelona, Spain; 6 Centro de Investigación Biomédica en Red de Cáncer (CIBERONC), Barcelona, Spain; Stanford University, UNITED STATES

## Abstract

The capped Small segment mRNA (SmRNA) of the *Andes orthohantavirus* (ANDV) lacks a poly(A) tail. In this study, we characterize the mechanism driving ANDV-SmRNA translation. Results show that the ANDV-nucleocapsid protein (ANDV-N) promotes *in vitro* translation from capped mRNAs without replacing eukaryotic initiation factor (eIF) 4G. Using an RNA affinity chromatography approach followed by mass spectrometry, we identify the human RNA chaperone Mex3A (hMex3A) as a SmRNA-3’UTR binding protein. Results show that hMex3A enhances SmRNA translation in a 3’UTR dependent manner, either alone or when co-expressed with the ANDV-N. The ANDV-N and hMex3A proteins do not interact in cells, but both proteins interact with eIF4G. The hMex3A–eIF4G interaction showed to be independent of ANDV-infection or ANDV-N expression. Together, our observations suggest that translation of the ANDV SmRNA is enhanced by a 5’-3’ end interaction, mediated by both viral and cellular proteins.

## Author summary

*Andes orthohantavirus* (ANDV) is endemic in Argentina and Chile and is the primary etiological agent of hantavirus cardiopulmonary syndrome (HCPS) in South America. ANDV is unique among other members of the *Hantaviridae* family of viruses because of its ability to spread from person to person. The molecular mechanisms driving ANDV protein synthesis remain poorly understood. A previous report showed that translation of the Small segment mRNA (SmRNA) of ANDV relied on both the 5’cap and the 3’untranslated region (UTR) of the SmRNA. In this new study, we further characterize the mechanism by which the 5’ and 3’end of the SmRNA interact to assure viral protein synthesis. We establish that the viral nucleocapsid protein N and the cellular protein hMex3A participate in the process. These observations indicated that both viral and cellular proteins regulate viral gene expression during ANDV infection by enabling the viral mRNA to establish a non-covalent 5’-3’end interaction.

## Introduction

*The Andes orthohantavirus* (ANDV), a rodent-borne member of the *Hantaviridae* family of the *Bunyavirales* order, is the etiological agent of hantavirus cardiopulmonary syndrome (HCPS) in Argentina and Chile [1]. ANDV mainly infects humans through aerosolized excreta and secretions from infected long-tailed pygmy rice rat (*Oligoryzomys longicaudatus*). A unique feature of ANDV is that its infection can also occur through person-to-person transmission [2,3].

The ANDV genome consists of three negative polarity single-stranded RNA segments, large (L), medium (M), and small (S), packed into helical nucleocapsids [4]. Transcription of the genomic RNA segments yields the 5’-7-methylguanosine (m^7^GTP) capped L, M, and S messenger RNAs (mRNAs). The mRNAs of *orthohantavirus* acquire their 5’cap- through a cap-snatching mechanism occurring in cytoplasmic processing bodies (P bodies) [5]. Interestingly, unlike most cellular mRNAs, the ANDV SmRNA and the LmRNA lack 3’poly(A) tails [6]. The LmRNA encodes a viral RNA-dependent RNA polymerase required for viral RNA transcription and replication [7,8]. The MmRNA encodes the glycoprotein precursor, which is cotranslationally processed, generating Gc and Gn, the two viral envelope glycoproteins [9]. The SmRNA encodes for the nucleocapsid protein (N) and the nonstructural S segment (NSs) protein [10,11]. Interestingly, the N-protein of other *orthohantavirus* participates in viral mRNA translation [12].

Eukaryotic mRNA translation comprises four stages initiation, elongation, termination, and ribosome recycling [13–16]. Translational control is exerted mainly at the initiation stage [14,15]. Translation initiation involves the eukaryotic initiation factor (eIF) 4F recognizing the mRNAs 5’cap, recruitment of the 40S ribosomal subunit, 5’-3’scanning of the mRNA until encountering the initiation codon, and the recruitment of the 60S ribosomal subunit [15–17]. Translation initiation ends with the 80S ribosome assembled and positioned at the initiation codon [15–17]. Eukaryotic mRNAs feature a 5’cap, and most have a 3’poly(A) tail [18–21]. The 5’cap and the 3’poly(A) tail establish a non-covalent interaction (closed-loop) that promotes efficient mRNA translation initiation, translation re-initiation through ribosome recycling, and enhanced mRNA stability [20,22,23]. For most eukaryotic mRNAs, the 5’-3’end interaction is mediated by the association of the capped-bound eIF4F with the poly(A)-bound poly(A)-binding protein (PABP) [20,24,25]. The heterotrimeric eIF4F protein complex comprises the cap-binding protein eIF4E, the RNA-dependent ATPase/RNA helicase eIF4A, and the high-molecular-weight scaffolding protein eIF4G [15–17]. Thus, the non-covalent mRNA circle is closed by eIF4G, which simultaneously interacts with the mRNA 5’capped-bound eIF4E and the 3’poly (A)-bound PABP [20,24]. In cells, assembly of the eIF4F complex and establishing the 5’cap-3’poly(A) interaction are tightly regulated processes [13,20].

In this study, we were interested in evaluating if the ANDV-N could replace the function of eIF4F, as described for other *orthohantavirus* [12]. Also, we sought to further understanding how translation of the ANDV SmRNA overcomes the need for a poly(A) tail. Consistent with earlier reports from other *orthohantavirus* N proteins [12,26], we show that the ANDV-N stimulates mRNA translation in a 5’cap dependent fashion. However, in contrast to the N of other *orthohantavirus* [12], the ANDV-N could not substitute all functions of eIF4F. Data also confirm previous reports showing that the 3’UTR of the ANDV SmRNA plays an essential role during viral mRNA translation [27]. Furthermore, we report that the human RNA-binding protein Mex3A (hMex3A) binds to the ANDV SmRNA 3’UTR, promoting translation of the SmRNA. The ANDV-N and hMex3A additively stimulate translation from the virus-like SmRNA in cells. However, results show that the ANDV-N and the hMex3A do not interact. Nonetheless, both proteins independently interact with eIF4G. Thus, this study provides novel insights regarding the role of cellular and viral proteins in supporting ANDV SmRNA poly(A)-independent translation.

## Results

### ANDV-N protein stimulates cap-dependent translation initiation

**Fig 1:**
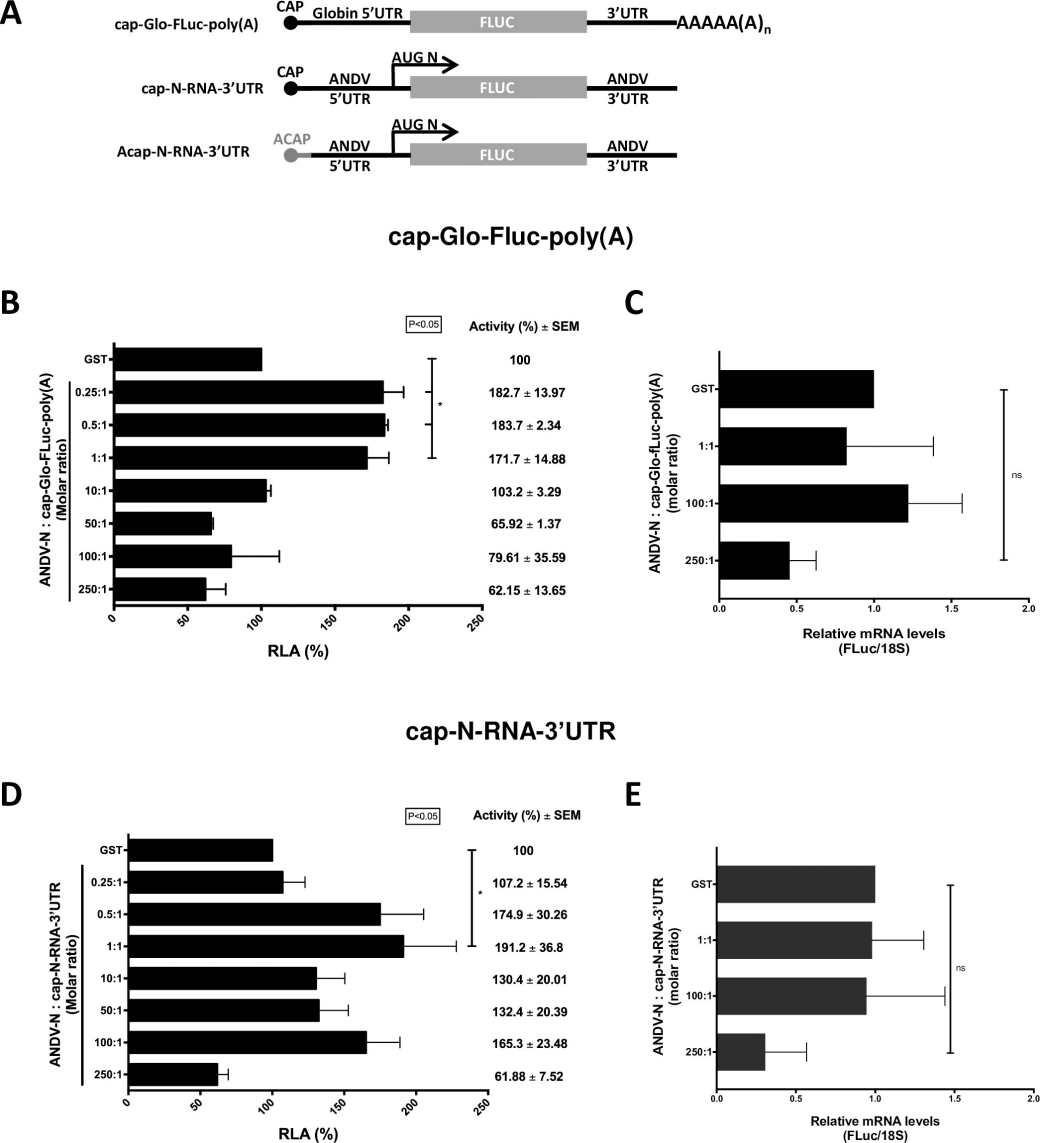
*In vitro* translation of ANDV-like SmRNAs in the presence of partially purified recombinant ANDV GST-N protein. **(A)** Schematic representation of the capped and polyadenylated Glo-FLuc RNA and capped ANDV like SmRNA, N-RNA-3’UTR [27], used in this study. In the ANDV like SmRNA RNAs, the FLuc reporter gene is in-frame with the ANDV-N protein initiation codon (depicted by the arrow) [27]. **(B-E)** *In vitro* translation (90 min at 30°C) in nuclease-treated Flexi-rabbit reticulocyte lysate (RRL; 35% v/v) programmed with *in vitro* transcribed cap-Glo-FLuc-poly(A) (B-C), or the cap-N-RNA-3’UTR (D-E) RNAs (0.01 pmol) was conducted in the presence of increasing concentrations of the ANDV GST-N protein (protein/RNA molar ratio from 0.25:1 to 250:1). GST alone at a protein/RNA molar ratio of 1:1 was used as a control. Data are shown as ratios, the number of ANDV GST-N protein molecules added to each reaction, relative to the estimated number of cap-N-RNA-3’UTR molecules added to the reaction (6.02 x10^9^ molecules/μL). The number of RNA molecules present in 0.01 pmol (6.02 x10^9^ molecules) was estimated considering an RNA length of 2286 bp for cap-N-RNA-3’UTR, and 2086 bp for the cap-Glo-FLuc-poly(A) RNAs, using the free online calculator [http://www.molbiol.ru/eng/scripts/01_07.html]. The number of molecules of GST (35kDa) or the recombinant ANDV GST-N (77.5 kDa) added to the reaction was estimated using the free online calculator [https://www.bioline.com/us/media/calculator/01_04.html]. **(C and E)** After the in vitro translation assay was performed 1μL of the reaction mix was taken from the indicated experimental points and diluted (1/100) and directly used as a template for a real-time RT-qPCR reaction to determine the relative levels of cap-Glo-FLuc-poly(A) RNA (C) and cap-N-RNA-3’UTR (E) RNA. The RNA abundance was expressed relative to the value obtained for the RRL supplemented only with GST at a 1:1 RNA to protein ratio set to 1. Values are the mean (+/- SEM) of at least three independent experiments, each conducted in duplicate. Statistical analysis was performed by a one-way ANOVA with Dunnett’s multiple comparisons test (P<0.05).

The N protein of the *Sin Nombre orthohantavirus* (SNV) stimulates viral and non-viral mRNA translation *in vitro* and in cells [12,26]. This ability is shared by the N protein of other *Bunyavirales* order members [28]. Based on these findings, we sought to establish if the N of ANDV (ANDV-N) could stimulate *in vitro* mRNA translation. For this, we used two *in vitro* transcribed mRNAs, the capped (cap) and polyadenylated (poly(A)) Glo-FLuc RNA (Fig 1A) and the cap-N-RNA-3’UTR RNA [27]. In the cap-Glo-FLuc-poly(A) RNA, the globin mRNA 5’UTR is followed by the firefly luciferase (FLuc) open reading frame (ORF) [29,30]. In the cap-N-RNA-3’UTR RNA, the ANDV SmRNA 5’and 3’UTR flank the FLuc ORF in-frame with the N-protein initiation codon, AUG_N_ (Fig 1A) [27]. A GST-tagged version of the ANDV-N (ANDV GST-N) was expressed in bacteria, purified, concentrated, and added to the nuclease-treated Flexi-rabbit reticulocyte lysate (RRL) programmed with either the *in vitro* transcribed cap-Glo-FLuc-poly(A) or the cap-N-RNA-3’UTR RNAs. Luciferase activity was measured, and data were expressed as relative luciferase activity (RLA), with the FLuc activity obtained in RRL programmed with mRNA and GST alone, added in a 1:1 protein/RNA ratio set to 100%. Results showed that the addition of the ANDV GST-N protein to the RRL stimulated translation of both the cap-Glo-FLuc-poly(A) (Fig 1B) and the ANDV-like SmRNA (Fig 1D) RNAs. Translation of the cap-Glo-FLuc-poly(A) was significantly stimulated (~83% increase) at a 0.25:1 (protein:RNA) ratio (Fig 1B). The stimulatory effect of the ANDV GST-N protein was maintained up to the 1:1 (protein:RNA) ratio, after which translation returned to the GST-control levels (10:1 ratio) and even declined by 34% (not significant) at higher protein:RNA ratios (Fig 1B). To determine if the addition of GST or ANDV-GST N to the translation reaction had an impact on the stability of the cap-Glo-FLuc-poly(A) RNA, after the *in vitro* translation 1 μL of the final reaction mix (GST, 1:1, 100:1, and 250:1) was diluted (1/100) in nuclease-free water and directly used as a template for quantitative analysis by a real-time RT-qPCR. No significant changes in transcript levels between treatments (1:1 and 1:100 ratios) were detected, indicating that the variations in FLuc activity do not directly link with RNA levels (Fig 1C). However, a nonsignificant reduction of RNA levels was apparent at 250:1 protein to RNA ratios (Fig 1C). Thus, we cannot discard that the nonsignificant decrease in FLuc activity (~34% reduction) at 250:1 protein to RNA ratios (Fig 1B) could be due to RNA instability (Fig 1C). Translation of the ANDV-like SmRNA was also significantly stimulated (~91% increase) by ANDV-N but only at a 1:1 protein to RNA ratio (Fig 1D). However, a stimulatory trend was maintained up to 100:1 protein to RNA ratios, declining (not significantly) by 38% at the 250:1 ratio. As above, RNA levels were determined at the end of the in vitro translation (GST, 1:1, 100:1, and 250:1). Again, no significant changes in transcript levels between treatments were detected after the *in vitro* translation (Fig 1E). However, as for the cap-Glo-FLuc-poly(A) RNA (Fig 1C), a nonsignificant decrease in cap-N-RNA-3’UTR RNA levels was observed at 250:1 protein to RNA ratios (Fig 1E). So, as indicated, the relative reduction in FLuc activity (~38% reduction) at 250:1 protein to RNA ratios (Fig 1D) could be due to a decrease in the levels of RNA (Fig 1E). Taken together, the above results confirm that, like the N protein of other members of the *Bunyavirales* order [12,26,28], the ANDV-N stimulates translation (compare GST and 1:1 protein to RNA ratios) of both non-viral-like and viral-like mRNAs.

### *In vitro*, the ANDV-N protein stimulates translation in a cap-dependent manner but cannot replace a cleaved eIF4G

**Fig 2:**
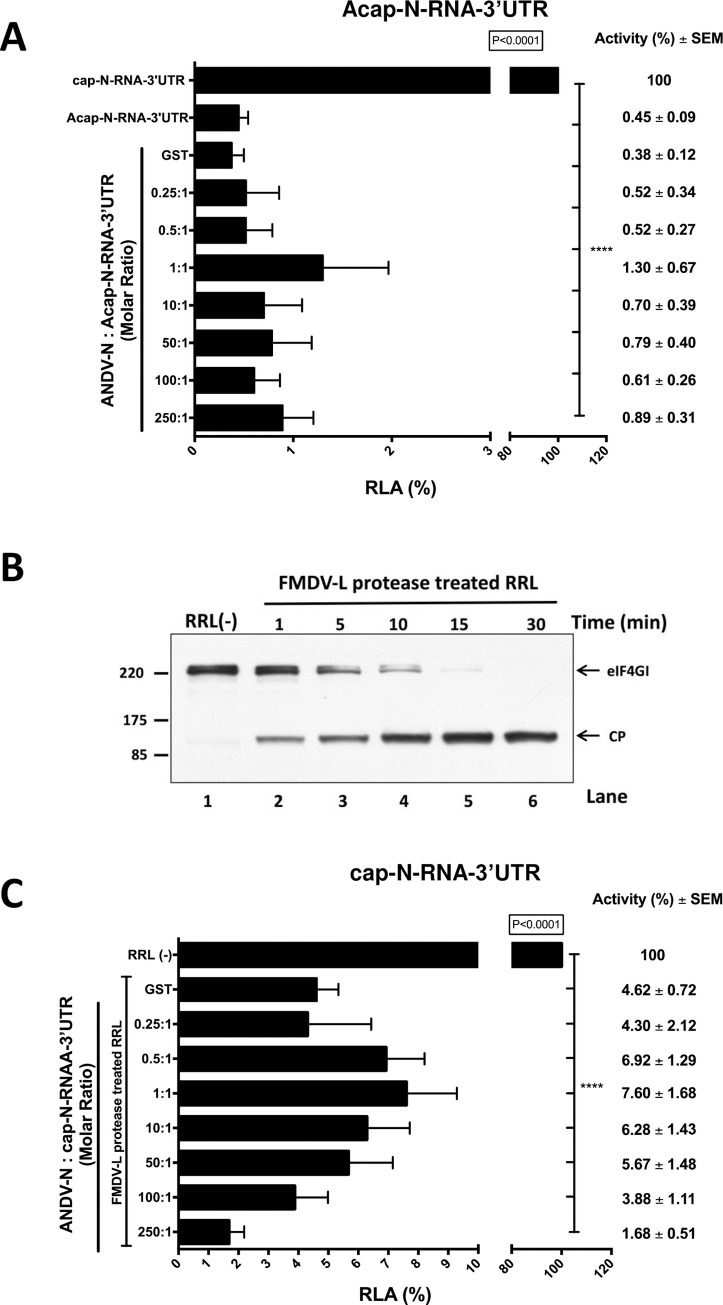
*In vitro*, the partially purified recombinant ANDV GST-N protein does not stimulate translation of an Acapped-RNA, nor does it substitute for eIF4G. **(A)***In vitro* translation of the cap-N-RNA-3’UTR and Acap-N-RNA-3’UTR (0.01 pmol) under similar conditions as described in (Fig 1). The Acap-N-RNA-3’UTR RNA was also translated in the presence of increasing concentrations of the ANDV GST-N protein (protein/RNA molar ratio from 0.25:1 to 250:1). GST alone at a protein/RNA molar ratio of 1:1 was used as a control. Data are shown as ratios, the number of ANDV GST-N protein molecules added to each reaction, relative to the estimated number of Acap-N-RNA-3’UTR molecules added to the reaction (see legend to Fig 1). **(B)** RRL (35% v/v) was supplemented with L-protease-RRL (4% v/v), RRL programmed with FMDV L protease RNA template, or not (lane 1), from 1 to 30 minutes (lanes 2 to 6). Western analysis was performed using polyclonal antibodies against eIF4GI [74]. Positions of molecular mass standards (in kDa) are shown. The cleavage product (CP) has been previously characterized [34]. **(C)** *In vitro* translation (90 min at 30°C) of RRL (35% v/v) treated for 30 min with L-protease-RRL (4% v/v) before the addition of the cap-N-RNA-3’UTR (0.01 pmol) RNA, in the presence of increasing concentrations of recombinant ANDV GST-N protein (0.5:1 to 250:1 protein to RNA ratio). Values are the mean (+/- SEM) of at least three independent experiments, each conducted in duplicate. Statistical analysis was performed by a one-way ANOVA with Dunnett’s multiple comparisons test (P<0.05).

Next, we sought to establish if the effect of ANDV-N on *in vitro* translation of the virus-like mRNA was cap-dependent. For this, the *in vitro* translation reactions were programmed with the cap-N-RNA-3’UTR or the ApppG-N-RNA-3’UTR RNAs [27]. The ApppG cap-analog (Acap) does not support efficient cap-dependent translation initiation as it is not recognized by eIF4E [31,32]. Thus, eIF4F cannot assemble on the mRNAs 5’end [31,32]. The *in vitro* translation reactions were supplemented as indicated above (Fig 1B and 1D) with GST, added in a 1:1 protein to RNA ratio (control), or increasing protein to RNA ratios of ANDV GST-N. Luciferase activity was measured, and data were expressed as RLA. The FLuc activity obtained with the capped-N-RNA-3’UTR RNA was set to 100%. In agreement with our previous report [27], the replacement of the 5’cap by an Acap hampered translation from the viral-like mRNA (~99% inhibition) (Fig 2A compare cap-N-RNA-3’UTR and Acap-N-RNA-3’UTR). Results showed that neither GST nor the recombinant ANDV GST-N could fully restore translation from the Acap-N-RNA-3’UTR RNA (Fig 2A). Together results indicated that *in vitro*, the ANDV GST-N protein stimulates translation of a virus-like SmRNA in a 5’cap-dependent manner.

In cells, the SNV-N protein can functionally replace the entire eIF4F complex, which includes eIF4E, eIF4A, and eIF4G [12]. Nonetheless, the N-protein of the Crimean-Congo hemorrhagic fever virus (CCHFV), a tick-borne Nairovirus within the *Bunyaviridae* family, requires eIF4G to stimulate translation [28]. So next, we focused on eIF4G and sought to determine if the ANDV-N protein could replace its function *in vitro*. For this, we evaluated if the ANDV-N protein could rescue SmRNA translation in RRL treated with the foot-and-mouth disease virus (FMDV) leader protease (L protease) [30,33–37]. The FMDV L protease cleaves eIF4G abrogating the interaction of cap-bound eIF4E with the rest of the eIF4F complex, leading to the specific inhibition of cap-dependent translation initiation [33,38,39]. The FMDV L protease was synthesized in RRL as previously described [30,35–38]. As a control, an *in vitro* translation reaction lacking the FMDV L protease encoding RNA was conducted in parallel. The FMDV L protease RRL, or the control RRL, was then added (4% v/v) to fresh RRL. The cleavage of endogenous eIF4G was monitored through time (1 to 30 min) by western blot (Fig 2B, lanes 2 to 6). The RRL(-) corresponds to a 30 min control reaction (Fig 2B, lane 1). Results showed that at 30 min post-treatment, full-eIF4G cleavage was attained only in RRL treated with the FMDV L protease (Fig 2B, lane 6). RRL(-) or FMDV L protease treated RRL (30 min) were then programmed with cap-N-RNA-3’UTR RNA in the absence or the presence of increasing concentrations of ANDV-N protein. Luciferase activity was measured, and data expressed as RLA, with the FLuc activity obtained in RRL(-) set to 100%. Consistent with our previous report [27], translation of the cap-N-RNA-3’UTR RNA was significantly reduced (~94% inhibition) in RRL treated with the FMDV-L protease when compared with RRL(-) (Fig 2C). This observation confirmed that in the absence of viral proteins, translation of the cap-N-RNA-3’UTR RNA requires the eIF4E-eIF4G interaction [27]. The addition of GST or ANDV-GST N protein (~92% inhibition; 1:1 protein /RNA) to the FMDV L protease treated RRL did not restore translation from the cap-N-RNA-3’UTR RNA (Fig 2C). Based on these observations, we conclude that in RRL, the ANDV GST-N protein could not replace the function of eIF4G.

### The ANDV-N protein stimulates translation of an ANDV-like mRNA in cells

**Fig 3:**
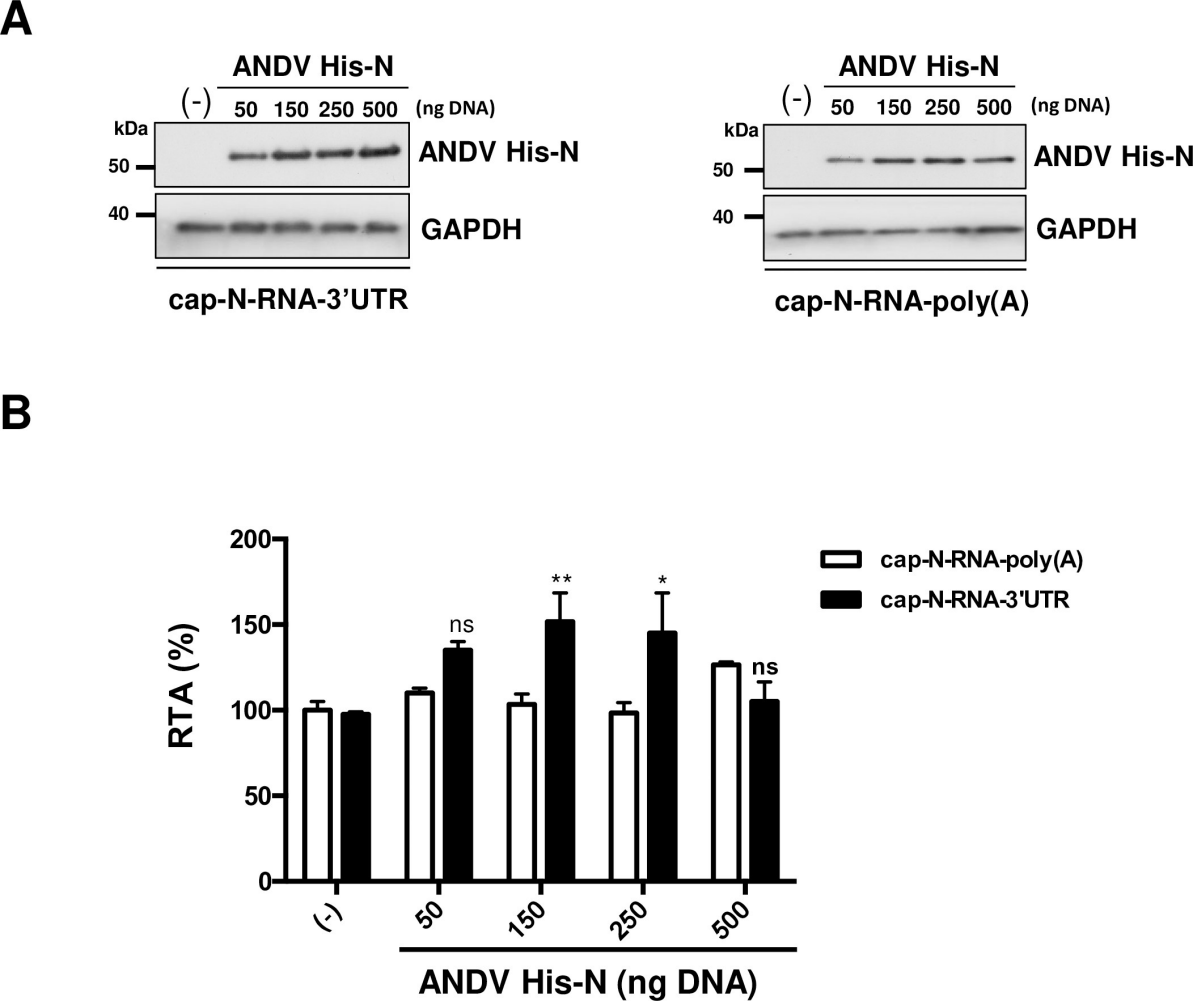
The ANDV-N protein enhances translation of the ANDV-like SmRNA in cells. HEK-293T cells were transfected with increasing amounts (50–500 ng) of a plasmid encoding for the recombinant ANDV His-N protein. Total transfected DNA was kept constant at 500 ng using pSP64 Poly(A) DNA. *In vitro* transcribed cap-N-RNA-3’UTR or cap-N-RNA-poly(A) RNAs, were transfected in cells together with a capped and polyadenylated mRNA encoding for RLuc (RLuc RNA). Six hours post-transfection (hpt), cells were lysed, and FLuc and RLuc activities were measured. **(A)** Expression of the ANDV His-N protein was confirmed by western blotting, using a monoclonal mouse anti-His antibody. GAPDH was used as a loading control. (**B)** The FLuc and RLuc activities were used to determine the relative translation efficiency (RTA), FLuc/RLuc, for each mRNA. Values shown are the mean (+/- SEM) of at least three independent experiments, each conducted in triplicate. Statistical analysis was performed by a two-way ANOVA with Sidak’s multiple comparisons test (P<0.05).

Next, we wondered whether the ANDV-N protein could stimulate the translation of the virus-like SmRNA in cells. For this, HEK 293T cells were transfected with a plasmid encoding a His-tagged ANDV-N protein (ANDV His-N). Forty-eight hours after, cells were transfected with the cap-N-RNA-3’UTR or the cap-N-RNA-poly(A) RNAs together with a capped and polyadenylated *Renilla* luciferase (RLuc) encoding mRNA (used as a control for RNA transfection efficiency). In the cap-N-RNA-poly(A) RNA, the ANDV SmRNA 3’UTR was substituted by a poly(A) tail [27]. Six hours post-RNA transfection, cells were lysed, and luciferase activities were determined and normalized to the RLuc control. Expression of the ANDV His-N protein was monitored by western blotting, using an anti-His antibody. In these assays, the glyceraldehyde 3-phosphate dehydrogenase (GAPDH) was used as a loading control (Fig 3A). FLuc activity was normalized to RLuc activity (FLuc/RLuc ratio), and results are presented as relative translation efficiency (RTA). The FLuc/RLuc ratios obtained for the cap-N-RNA-3’UTR and the cap-N-RNA-poly(A) RNAs alone were set to 100%. Results showed that in cells, expression of the ANDV His-N protein stimulated FLuc synthesis from the cap-N-RNA-3’UTR mRNA in a concentration-dependent manner, without impacting the translation of the cap-N-RNA-poly(A) mRNA (Fig 3B). These results suggest that in cells, translation stimulation of the virus-like RNA induced by the ANDV His-N protein depends on the presence of the 3’UTR of viral SmRNA.

### The ANDV-N protein interacts with the eIF4G in cells

**Fig 4:**
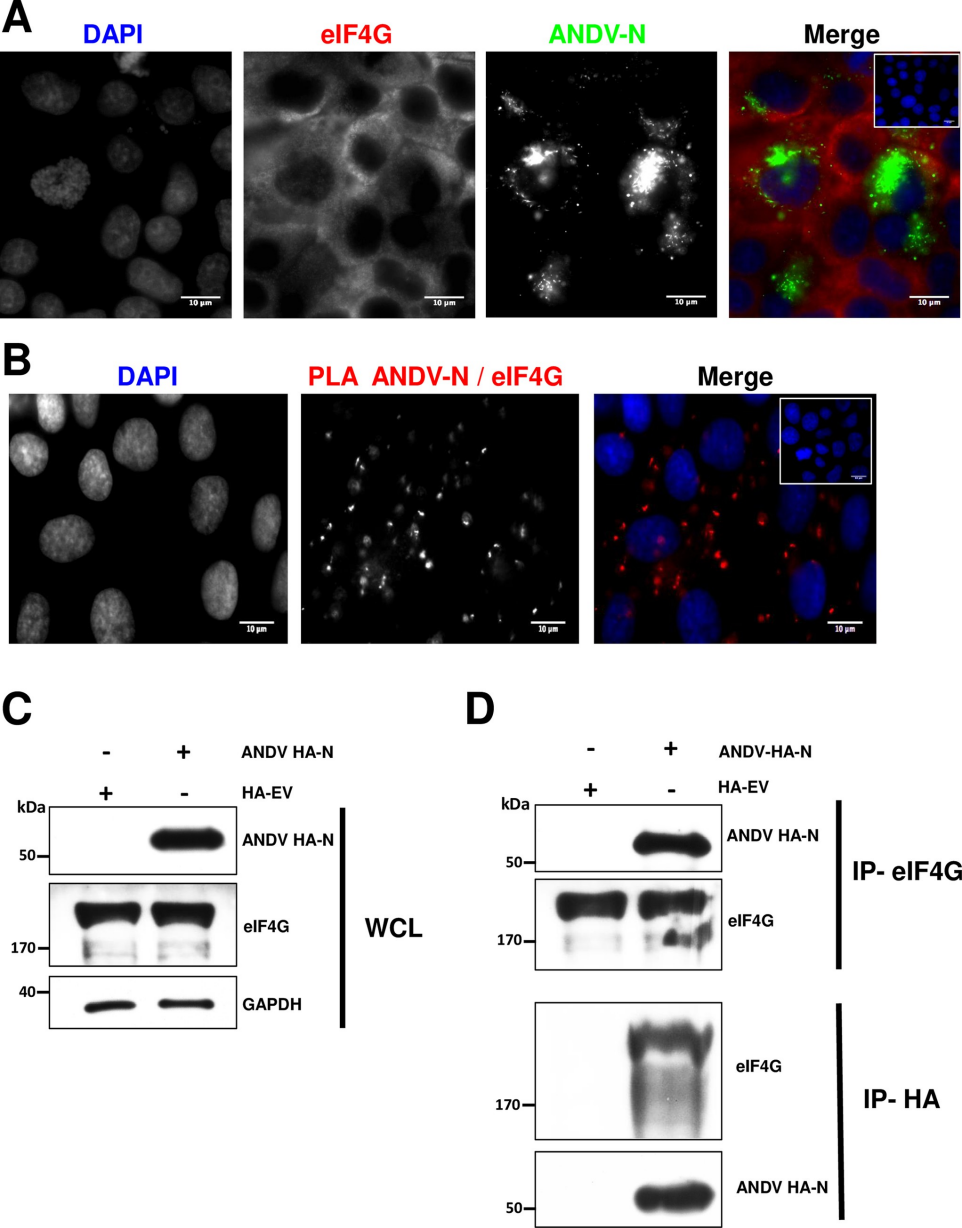
The ANDV-N protein and eIF4G interact in ANDV infected Huh-7 cells. **(A)** Huh-7 cells were infected with ANDV (MOI 1), and 24 hours post-infection (hpi), cells were fixed (PFA 4%) and permeabilized (PBS-Triton). Expression of the ANDV-N and endogenous eIF4G proteins was confirmed by immunofluorescence (IF) using mouse monoclonal anti-ANDV-N or rabbit polyclonal anti-eIF4G as primary antibodies. As secondary antibodies, an Alexa 488 donkey anti-mouse or an Alexa 594 donkey anti-rabbit was used, respectively. As a negative control for the IF, the inset, in the upper right corner of the merge, shows ANDV infected cells where detection was conducted using only the secondary antibody. **(B)** ANDV-infected cells were incubated with primary antibodies as in (A), but PLA secondary antibodies were used following the manufacturer’s instructions. The inset, in the upper right corner of the merge, shows infected cells without primary antibodies but with the PLA secondary antibodies added as a negative control of PLA. Vectashield with DAPI was used as mounting media. The images were obtained in Olympus epifluorescence Microscope and processed by Image J. **(C-D)** HEK293T cells were cotransfected with the ANDV HA-N or HA-EV plasmids. Forty-eight hours later, cells were lysed, and immunoprecipitation (IP) assays were performed using protein A/G agarose coated with the corresponding antibody. The beads were washed and incubated with loading buffer at 95°C and the supernatant, which was used for western blotting. **(C)** Whole-cell lysate (WCL) of each sample and **(D)** IP fractions with the corresponding primary antibody highlighted on the right side of the panels. Western blotting was performed using mouse anti-HA, rabbit anti-eIF4G, and mouse anti-GAPDH. Horseradish Peroxidase (HRP)-conjugated Protein A/G was used to detect the primary antibodies.

Next, we wondered if, during infection, the ANDV-N and eIF4G proteins interacted in cells. For this, Huh-7 cells were infected with the ANDV CHI-7913 isolate (MOI of 1). Twenty-four hours post-infection, the expression of the ANDV-N and the endogenous eIF4G protein was evaluated by immunofluorescence (IF) (Fig 4A). The endogenous eIF4G protein showed a diffuse cytoplasmic distribution, while the distribution of the ANDV-N was also cytoplasmic but predominantly peri-nuclear (Fig 4A). We then performed an *in situ* proximity ligation assay (PLA) [40,41], using a rabbit anti-eIF4G and a mouse anti-ANDV-N as primary antibodies (Fig 4B). PLA recognizes target molecules in close proximity (<40 nm), and a positive signal is considered to reflect a protein-protein interaction [40,41]. ANDV-infected cells developed using only the secondary antibodies were used as negative controls (Fig 4A and 4B, panel’s upper right corner). The PLA signal (red spots) suggests that in ANDV-infected Huh-7 cells, the ANDV-N protein and the endogenous eIF4G interact (Fig 4B).

Next, the above experiment was repeated in a virus-free environment. For this, we carried out reciprocal co-immunoprecipitation (Co-IP) experiments in HEK 293T cells transfected with ANDV HA-N expression plasmid or the HA-EV control plasmid. Forty-eight hours later, cells were lysed (whole-cell lysate, WCL), and the expression of ANDV HA-N, and endogenous eIF4G was confirmed by western blotting using GAPDH as a loading control (Fig 4C). WCL were then subjected to immunoprecipitation using an anti-eIF4G or anti-HA antibody. Results show that the ANDV HA-N Co-IP with eIF4G when anti-eIF4G was used (Fig 4D). Likewise, eIF4G Co-IP with ANDV HA-N when the anti–HA antibody was used (Fig 4D). These results confirm that the ANDV-N and eIF4G interact in cells.

### The human Mex 3A interacts with the 3’UTR of the ANDV SmRNA

**Fig 5:**
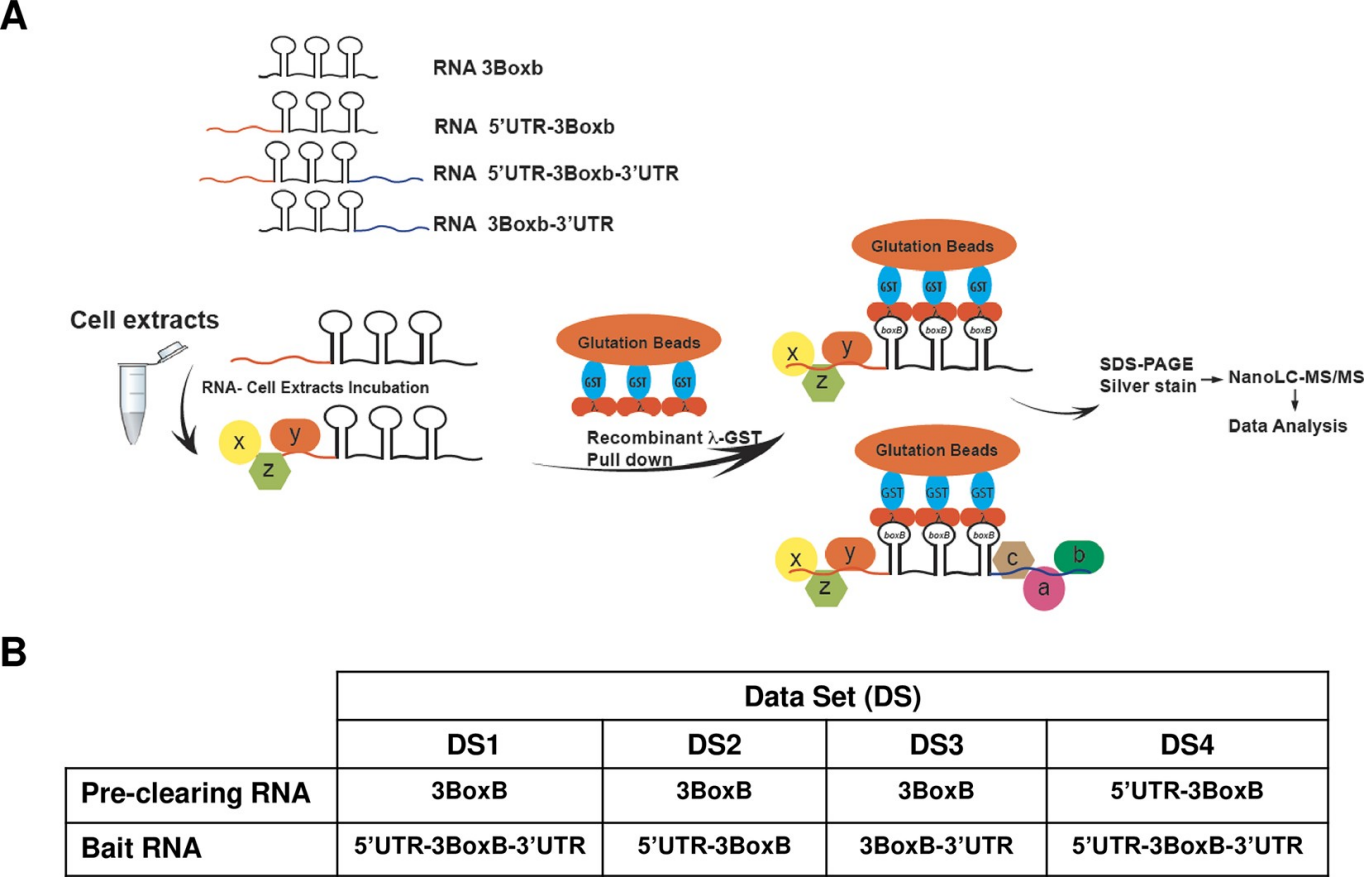
Identifications of host proteins that interact with the UTRs of the ANDV SmRNA. **(A)** Depiction of the GRNA affinity chromatography combined with nanoscale liquid chromatography coupled to tandem mass spectrometry (nano LC-MS/MS) strategy used to identify host proteins that interact with the 3’UTR of the ANDV SmRNA. **(B)** HeLa extracts precleared with 3BoxB, or 5’UTR-3BoxB RNA were incubated with different *in vitro* transcribed bait RNA. The RNA-proteins complexes were pulled down using recombinant λ-GST protein as described in [42]. The proteins bound to 3BoxB-3’UTR, 5’UTR-3BoxB, 5’UTR-3BoxB-3’UTR RNAs identified by Nano LC-MS/MS (Applied Biomics, Inc., Hayward, CA, USA) were grouped into four datasets (DS, the list of proteins can be found in S1 Data).

Previously [27], we showed that the 3’UTR of the ANDV SmRNA participates in cap-dependent translation of the virus-like SmRNA. These experiments, conducted in HeLa cells and in the absence of viral proteins, suggested that the 3’UTR played a role in the translation of the virus-like SmRNA [27]. Based on these findings, we predicted that in HeLa cells, at least one cellular protein was involved in the process of 5’-3’ end communication of the virus-like SmRNA [27]. So, using a GRNA affinity chromatography strategy combined with nanoscale liquid chromatography coupled to tandem mass spectrometry (nano LC-MS/MS) [42], we set out to identify proteins from HeLa cells that interact with the 3’UTR of the ANDV SmRNA (Fig 5). Briefly, using the ANDV SmRNA 5’and 3’UTRs, the 5’UTR-3BoxB, 5’UTR-3BoxB-3’UTR, and 3BboxB-3’UTR RNAs were generated (Fig 5A). An RNA containing only the tandem repeats of the BoxB sequence, the 3BoxB-RNA, was used as a control. HeLa cell extracts, used as a source of human proteins, were clarified from non-specific RNA-binding proteins (RBPs) or 5’UTR-binding proteins by incubation with an excess of 3BoxB or 5’UTR-3BoxB RNAs (Fig 5B). The 3BoxB-RNA-protein and the 5’UTR-3BoxB-protein complexes were recovered using bacteriophage λ anti-terminator protein (λN)-GST fusion protein bound to glutathione sepharose [42]. The recovered clarified-cell extracts were incubated with the 5’UTR-3BoxB-3’UTR, 5’UTR-3BoxB, or 3BoxB-3’UTR- RNAs (Fig 5A and 5B). RNA-protein complexes were isolated using λN-GST fusion protein bound to glutathione sepharose. The RNA-bound proteins were then identified by Nano LC-MS/MS (Applied Biomics, Inc., Hayward, CA, [https://www.appliedbiomics.com/]). Proteins associated with the 5’UTR-3BoxB, 5’UTR-3BoxB-3’UTR, and 3BoxB-3’UTR RNAs were grouped into four datasets (Fig 5B and S1 Data). A substantive strategy was applied to the datasets (DS) to identify proteins that preferentially bound the 3’UTR of the ANDV SmRNA (S1 Data). The rationale considered that proteins preferentially bound the SmRNA 3’UTR should be present in DS3, DS4, and DS1 but not in DS2 (Fig 5B). The initial analysis considered only DS3 and DS4 and their shared proteins (S1 Data, DS3-DS4). From the list of DS3 and DS4 shared proteins, the Neuronal acetylcholine receptor subunit alpha-10 (Accession number ACH10_HUMAN) and the Zinc finger protein 169 (Accession number ZN169_HUMAN) were also present in DS2 but not in DS1 (S1 Data). Therefore, these proteins were not considered for further analysis. Most of the other proteins shared in DS3 and DS4 such as Ras GTPase-activating-like protein IQGAP1 (Accession number IQGA1_HUMAN), Uncharacterized protein C2orf77 (Accession number CB077_HUMAN), Neuroblastoma-amplified sequence (Accession number NBAS_HUMAN), and others were present in all DS (1 through 4; see S1 Data). These proteins were also discarded for further analysis. The DDB1-CUL4-associated factor 5 (Accession number DCAF5_HUMAN), member of the cullin-RING ubiquitin ligases (CRLs) family of ubiquitin E3 ligases [43], and the human RNA chaperone protein Mex3A (hMex3A), an RBP that contains two K homology (KH) domains and a C-terminal RING finger module [44], were the only proteins to fulfill all selection criteria (S1 Data; present in DS3, DS4, and DS1, but not in DS2). The DNA excision repair protein ERCC-6-like (Accession number ERC6L_HUMAN), protein SIX6OS1 (Accession number S6OS1_HUMAN), and the E3 ubiquitin-protein ligase UBR3 (Accession number UBR3_HUMAN) that were present in DS3 and DS4 but not DS1 (S1 Data), were also good candidate 3’UTR-binding proteins, however, were arbitrarily omitted from the study.

**Fig 6:**
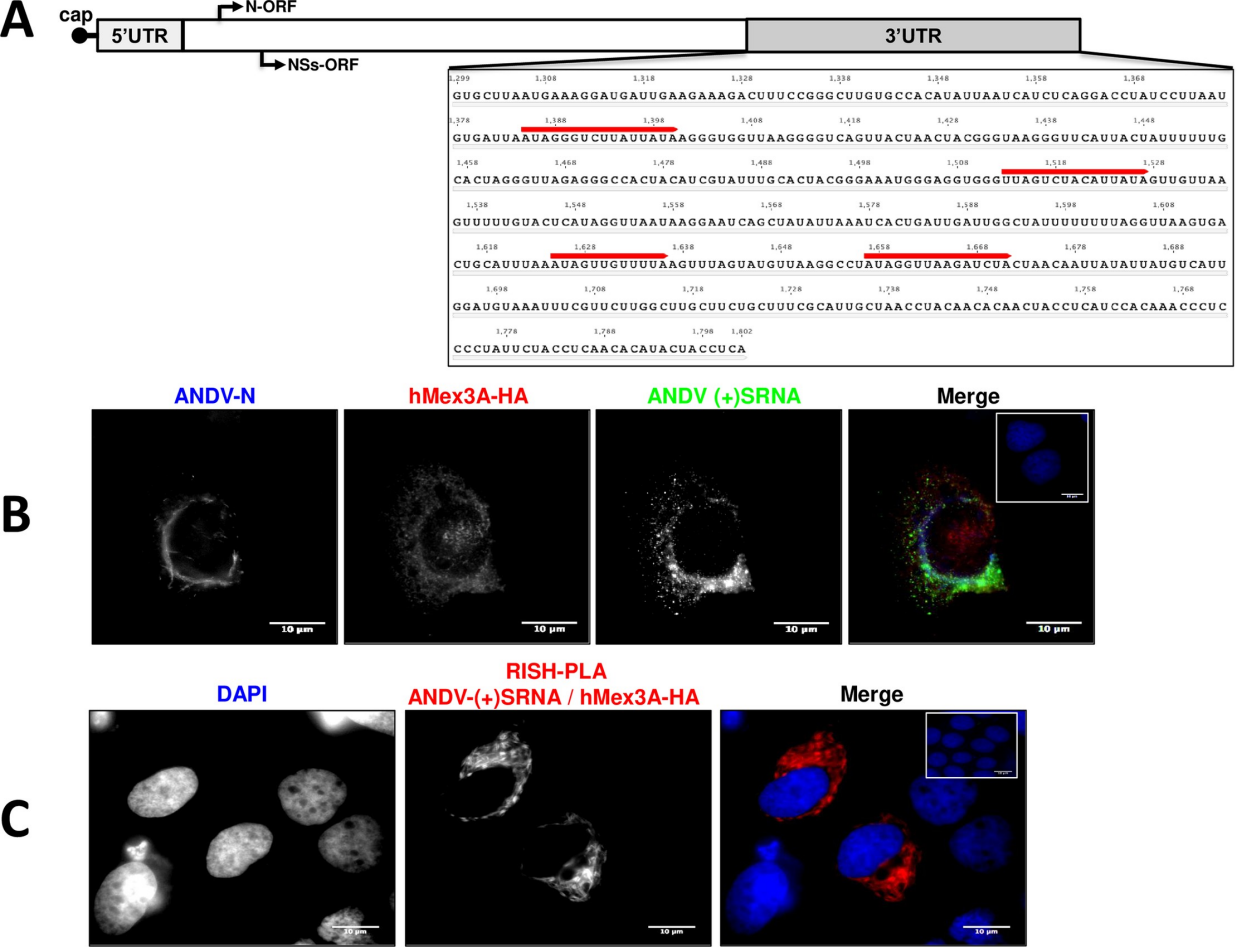
The recombinant human HA-Mex3A protein interacts with the ANDV SmRNA in ANDV infected Huh-7 cells. **(A)** Schematic representation of the SmRNA of ANDV isolate CHI-7913 indicating the 5’cap, 5’UTR, and the N and NSs translation initiation sites (black arrows). The sequence of the 3’UTR (nts 1299–1802; accession number AY228237.1) highlighting the Mex3A binding sites (red line), (A/G/U)(G/U)AGN_(0–8)_U(U/A/C)UA [45], identified by a DNA pattern search using Gene Infinity web bench (http://www.geneinfinity.org/sms/sms_DNApatterns.html). **(B)** Huh-7 cells were infected with ANDV (MOI 1), and 24 hpi transfected with a plasmid encoding for hMex3A-HA protein. Twenty-four hpt cells were fixed (PFA 4%) and permeabilized (PBS-Triton). Cells were incubated (formamide at 42°C for 16 hrs) with a riboprobe complementary to (+)ANDV-SRNA labeled with Digoxigenin (Dig). The cells were washed and incubated with mouse anti-ANDV-N, rabbit anti-HA, and sheep anti-Dig, as primary antibodies. An Alexa 405 donkey anti-mouse, Alexa 594 donkey anti-rabbit, or an Alexa 488 donkey anti-sheep were used as secondary antibodies. As a negative control of the fluorescence in situ hybridization (FISH)-Immunofluorescence (IF) assay, the inset, in the merge’s upper right corner, shows ANDV-infected and hMex3A-HA expressing cells developed using only the Alexa secondaries antibodies. **(C)** ANDV-infected and hMex3A-HA expressing cells were incubated with mouse anti-Dig ((+)SRNA) and rabbit anti-HA as primary antibodies. PLA secondary antibodies were used following the manufacturer’s instructions. As a negative control of PLA, the inset, in the upper right corner of the merge, shows cells developed with only the PLA secondary antibodies.

Several reported observations made hMex3A the most exciting candidate to further study. The hMex3A ortholog in *Caenorhabditis elegans* acts as a translational regulator that exerts its function by binding to the 3’UTR of its target mRNAs [45]. In cells, hMex3A plays a relevant role in the regulation of gene expression. For example, hMex3A binds to and stabilizes CDK6 and TCF7 mRNA levels [46], while it binds to and destabilizes the LAMA2 mRNA [47]. In addition, the hMex3A binds to the 3’UTR of CDX2 encoding mRNA, repressing its translation [48], and affects protein and mRNA levels of CCL2 [49]. In cells, hMex3A localizes to P-bodies [44,48,50], a site essential for *orthohantavirus* mRNA synthesis [5]. Also, Mex3 recognition elements (MRE) ((A/G/U)(G/U)AGN(0–8)U(U/A/C)UA [45]) were identified in the SmRNA 3’UTR of different reported ANDV isolates (Accession numbers: KY659432.1, AF291702.1, NC_003466.1, AY228237.1, AF324902.1, MN258223.1, MN258224.1, MN850083.1, MN850086.1). The number of MRE varied among the SmRNAs 3’UTR of the different ANDV isolates. The analyzed sequences include the SmRNA 3’UTR of the viral ANDV CHI-7913 isolate (Accession numbers AY228237.1; nts 1299–1802) used herein in the infection assays (Fig 6A). In concordance with the absence of hMex3A in dataset DS2 (S1 Data), no MRE was identified in the 5’UTR or the SmRNA of the ANDV CHI-7913 isolate. Based on these observations, we decided to continue with the study of hMex3A.

Next, we sought to confirm that hMex3A interacted with the positive sense (+)ANDV-SRNA in the context of a viral infection. The (+)ANDV-SRNA population comprises the SmRNA and the Small complementary RNA (ScRNA), used during viral replication as a template to generate the negative-stranded genomic SRNA [7]. For this, Huh-7 cells were infected with ANDV CHI-7913 isolate (MOI of 1) and then transfected with a plasmid encoding an HA-tagged hMex3A protein. The expression of the ANDV-N protein (infection control) and the recombinant hMex3A-HA protein was followed by IF (Fig 6B). In agreement with previous reports [46,48], transfected hMex3A predominantly showed cytoplasmic staining (Fig 6B). The presence of the ANDV-SRNA in cells was confirmed by fluorescence in situ hybridization (FISH) (Fig 6B). The hMex3A-HA-(+)ANDV-SRNA interaction was evaluated by RNA *In-situ* hybridization Proximity Ligation Assay (rISH-PLA) [51,52]. Results from the rISH-PLA (red signal) confirmed the interaction between the (+)ANDV-SRNA and hMex3A-HA in ANDV-infected Huh-7 cells (Fig 6C). Thus, in ANDV-infected cells, the recombinant hMex3A-HA protein interacts with the (+)ANDV-SRNA.

### The human Mex 3A stimulates ANDV SmRNA translation

**Fig 7:**
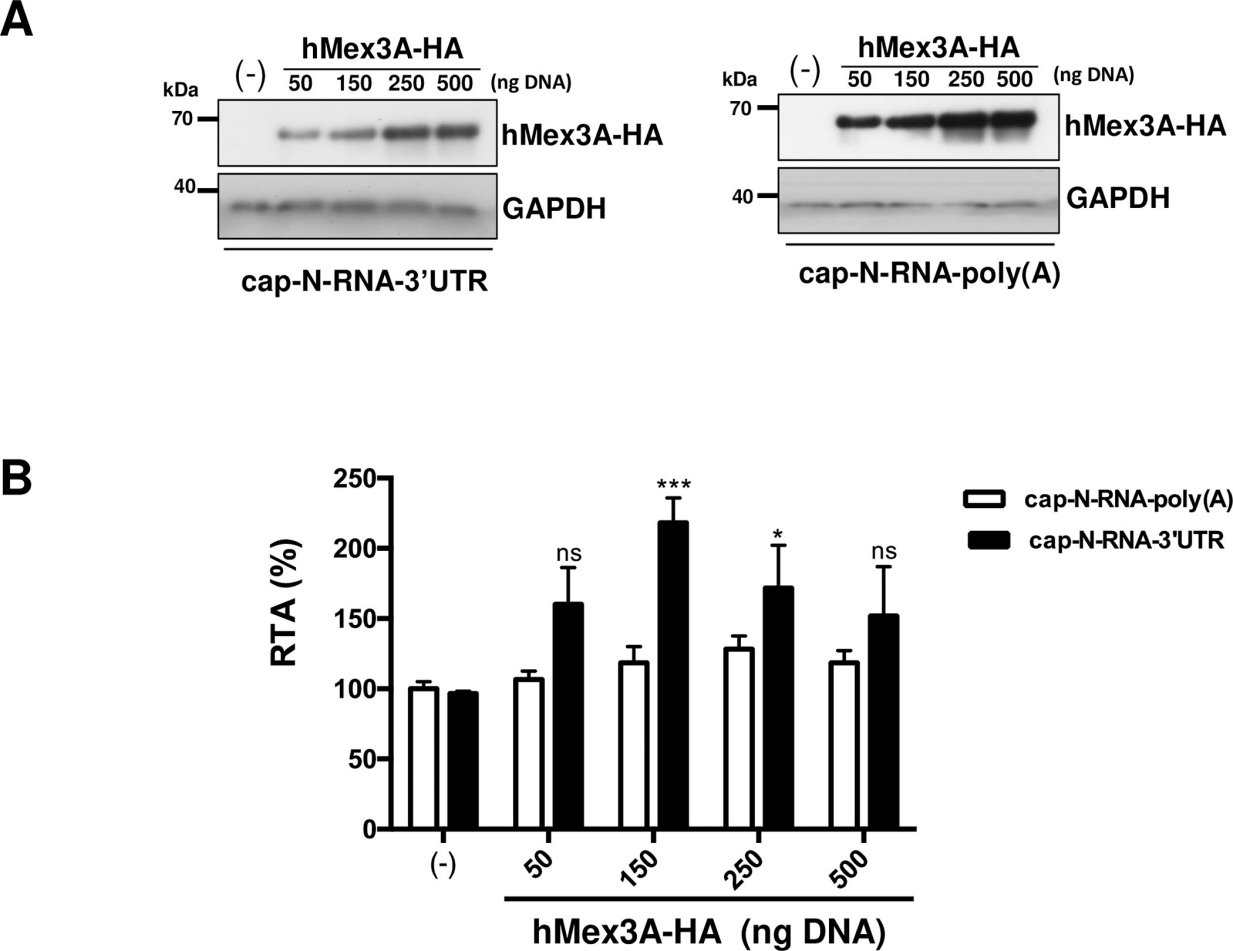
The recombinant HA-hMex 3A protein enhances translation of ANDV-like SmRNA in cells. HEK-293T cells were transfected with increasing amounts (50–500 ng) of a plasmid encoding for the recombinant hMex3A-HA protein. Total transfected DNA was kept constant at 500 ng using pSP64 Poly(A) DNA. The cap-N-RNA-3’UTR or cap-N-RNA-poly(A) RNAs were transfected in cells together with a capped and polyadenylated mRNA encoding for RLuc. Six hpt, cells were lysed, and FLuc and RLuc activities were measured. **(A)** Expression of the hMex3A-HA protein was confirmed by western blotting, using a monoclonal mouse anti-HA antibody. GAPDH was used as a loading control. (**B)** The FLuc and RLuc activities were used to determine the RTA for each mRNA. Values shown are the mean (+/- SEM) of at least three independent experiments, each conducted in triplicate. Statistical analysis was performed by a two-way ANOVA with Sidak’s multiple comparisons test (P<0.05).

Given that hMex3A-HA interacted with the (+)ANDV-SRNA in cells (Fig 6) and was identified as a SmRNA 3’UTR binding protein (S1 Data; DS3-DS4, DS1, and not in DS2), we next sought to evaluate if the protein participated in translation of the virus-like SmRNA in cells. For this, HEK 293T cells were transfected with a plasmid encoding the hMex3A-HA protein. Forty-eight hours later, cells were transfected with either the cap-N-RNA-3’UTR or the cap-N-RNA-poly(A) RNAs together with RLuc mRNA (used as a control for transfection efficiency). Six hours post mRNA transfection, the cells were lysed, and luciferase activities were determined and normalized to the RLuc control. The recombinant hMex3A-HA protein expression was confirmed by western blotting using an anti-HA antibody and detecting GAPDH as a loading control (Fig 7A). The FLuc/RLuc ratios obtained for the cap-N-RNA-3’UTR and the cap-N-RNA-poly(A) RNAs when used alone were set to 100%. In contrast, to what was reported for the cellular CDX2 mRNA [48], when overexpressed, the hMex3A-HA protein stimulated translation from the cap-N-RNA-3’UTR in a concentration-dependent manner (~120% increase at the maximum point) (Fig 7B). Interestingly, the expression of hMex3A-HA did not affect translation of the cap-N-RNA-poly(A) mRNA that lacks MREs (Fig 7B). Results showed that the impact of hMex3A-HA on the translation of the virus-like SmRNA was independent of the presence of viral proteins. Furthermore, results suggest that hMex3A-HA induced stimulation of the ANDV-like SmRNA was dependent on the presence of the ANDV SmRNA 3’UTR that harbors MREs (Fig 6A). This observation confirms that the 3’UTR of the SmRNA plays a role in translation, in this case, through the recruitment of at least one host protein.

### The hMex-3A protein stimulated ANDV-N protein-mediated translation

**Fig 8:**
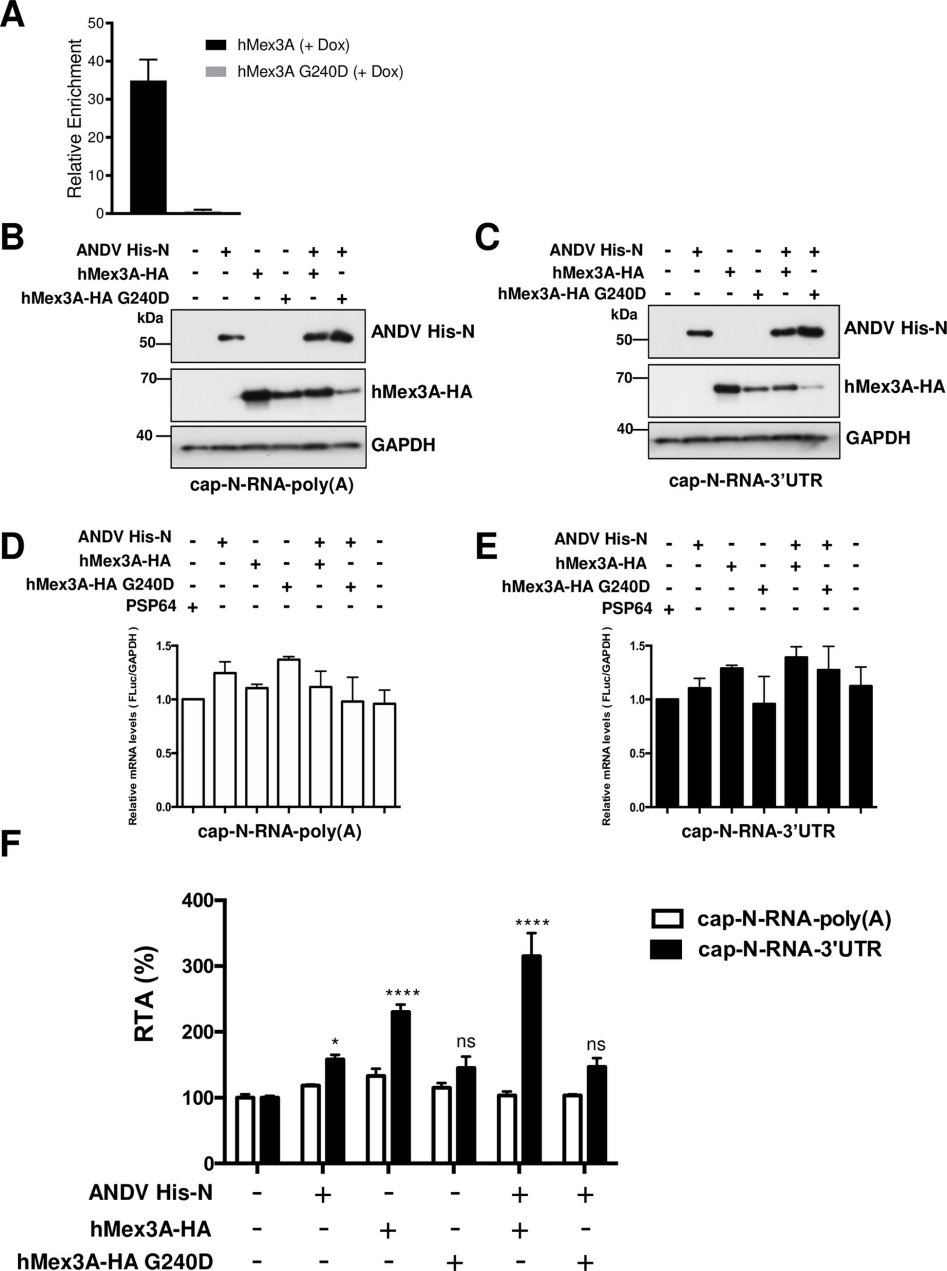
Coexpression of ANDV-N and hMex3A proteins additively stimulate translation from the ANDV-like SmRNA. **(A)** G240D mutation impairs the RNA binding of hMex3A. RT-qPCR of hMex3A bound EED in WT and G240D cells. LS174T cells bearing a doxycycline-inducible hMex3A -TAP or hMex3A G240D-TAP were induced (1 mg/mL) for 16 hours, and Ribonucleoprotein complex immune precipitation was performed to compare the binding of hMex3A WT and G240D mutant. The graph depicts the RIP vs. Input enrichment of the hMex3A target EED normalized to GAPDH. Bars show the mean and standard deviation of three (N = 3) independent RIP experiments. **(B-F)** HEK-293T cells were transfected with plasmids (150 ng) encoding for the recombinant ANDV His-N, hMex3A-HA, hMex3A-HA G240D proteins. Plasmids were transfected alone or in combination, keeping total transfected DNA constant at 500 ng using pSP64 Poly(A) DNA. The cap-N-RNA-3’UTR or cap-N-RNA-poly(A) RNAs were transfected in cells together with the RLuc RNA. Six hours post-transfection, cells were lysed, and FLuc and RLuc activities were measured. **(B-C)** Expression of the recombinant ANDV His-N, hMex3A-HA, and hMex3A-HA G240D proteins was confirmed by western blotting, using a monoclonal mouse anti- His or anti-HA antibody. GAPDH was used as a loading control. **(D-E)** Total RNA was extracted from cells transfected with the cap-N-RNA-poly(A) **(D)** or the cap-N-RNA-3UTR **(E)** RNAs together with the indicated DNA plasmids. The relative levels of FLuc encoding RNA and GAPDH mRNA were determined by real-time RT-qPCR. The normalized RNA levels (FLuc/GAPDH) were expressed relative to the value obtained in cells transfected with the pSP64 Poly(A) control DNA set to 1. Values are the mean ±SD of results from two independent experiments, each conducted in duplicate. Statistical analysis was performed by the ANOVA test followed by Dunnet multiple comparisons, *P **<** 0.01 versus the pSP64 Poly(A) DNA. **(F)** The FLuc and RLuc activities were used to determine the RTA (FLuc/RLuc ratio) for each mRNA. Values shown are the mean (+/- SEM) of at least three independent experiments, each conducted in triplicate. Statistical analysis was performed by a two-way ANOVA with Sidak’s multiple comparisons test (P<0.05).

The above data suggest that hMex3A binding to the 3’UTR is required for the protein to stimulate translation of the virus-like mRNA. To verify this hypothesis, we generated a hMex3A-HA RNA-binding mutant by introducing a G240D mutation in the first KH RBD of hMex3A by site-directed mutagenesis. To confirm that the introduced mutation was sufficient to hamper hMex3A’s ability to interact with its target RNA, a TAP-tagged version of the hMex3A G240D or the hMex-3A were cloned into a doxycycline-inducible lentiviral vector. The retroviral vectors and the helper plasmids encoding for the retroviral proteins were transfected into HEK 293T cells. Recombinant retroviral particles were harvested and used to transduce LS174T cells, which were then selected for stable integration of the recombinant DNA as indicated in Material and Methods. The Embryonic Ectoderm Development (EED) mRNA is a target of hMex3A in LS174T cells (F.M. Barriga and E. Batlle, personal communication). The hMex3A-TAP or hMex3A G240D-TAP protein expression was induced in LS174T cells, and a ribonucleoprotein complex immunoprecipitation (RIP) assay was conducted as previously described [53,54]. The amount of EED mRNA associated with the hMex3A-TAP and hMex3A G240D-TAP protein was assessed by RT-qPCR as indicated in Materials and Methods, and the differential RNA-binding was calculated. Results show that the G240D mutation introduced in the first KH RNA binding domain of hMex3A was sufficient to abolish the capacity of the protein to bind the EED mRNA in LS174T cells (Fig 8A). So, we conclude that the hMex3A G240D mutant protein has a reduced ability to bind its target RNA.

Next, HEK 293T cells were transfected with either the plasmid encoding for ANDV His-N, hMex3A-HA, the mutant hMex3A-HA G240D, or a combination of ANDV His-N with either hMex3A proteins. As a control, cells were transfected with the pSP64 PolyA or mock-transfected (No DNA) when indicated. Forty-eight hours later, cells were transfected with either the cap-N-RNA-3’UTR or the cap-N-RNA-poly(A) RNAs together with the RLuc mRNA (control for transfection efficiency). Six hours post-mRNA transfection, the cells were lysed, and the recombinant protein’s expression was confirmed by western blotting using GAPDH as a loading control (Fig 8B and 8C). In parallel, cytoplasmic RNA was extracted from transfected cells and used as a template for quantitative analysis of FLuc encoding mRNA and GAPDH mRNA by an RT-qPCR. This approach considered that the only source of FLuc encoding mRNA in the cells corresponds to the cap-N-RNA-poly(A) (Fig 8D) or the cap-N-RNA-3’UTR (Fig 8E) RNAs. No significant changes in the normalized (FLuc/GAPDH) transcript levels between treatments were detected for the cap-N-RNA-poly(A) (Fig 8D) or the cap-N-RNA-3’UTR RNAs (Fig 8E). Luciferase activities were determined and normalized to the RLuc control. The FLuc/RLuc ratios obtained for the cap-N-RNA-3’UTR and the cap-N-RNA-poly(A) RNAs in the absence of any other plasmid were set to 100%. Results, presented as RTA (%), confirmed that the individual expression of the ANDV His-N or the hMex3A-HA protein enhances translation from the N-RNA-3’UTR mRNA by ~58% and ~130%, respectively. Neither the expression of ANDV His-N or hMex3A-HA affected translation from the cap-N-RNA-poly(A) RNA (Fig 8F). These results confirmed that both protein’s actions over the viral-like SmRNA depend on the presence of the ANDV SmRNA 3’UTR. The co-expression of the ANDV His-N and hMex3A-HA additively increase (by ~215%) the rate of translation from only the cap-N-RNA-3’UTR mRNA with no evident impact on the cap-N-RNA-poly(A) mRNA (Fig 8F). The mutant hMex3A-HA G240D did not affect translation from either the cap-N-RNA-poly(A) or cap-N-RNA-3’UTR RNAs, nor was it capable of further enhancing ANDV-N mediated translation (Fig 8F). These findings suggest that hMex3A-HA binding to the ANDV SmRNA 3’UTR was required for the protein to stimulate translation from the ANDV SmRNA. Thus, results suggest that the 3’UTR recruits cellular proteins, which, together with viral proteins, are required for the optimal viral gene expression.

### The hMex3A protein interacts with the eIF4G in cells

**Fig 9:**
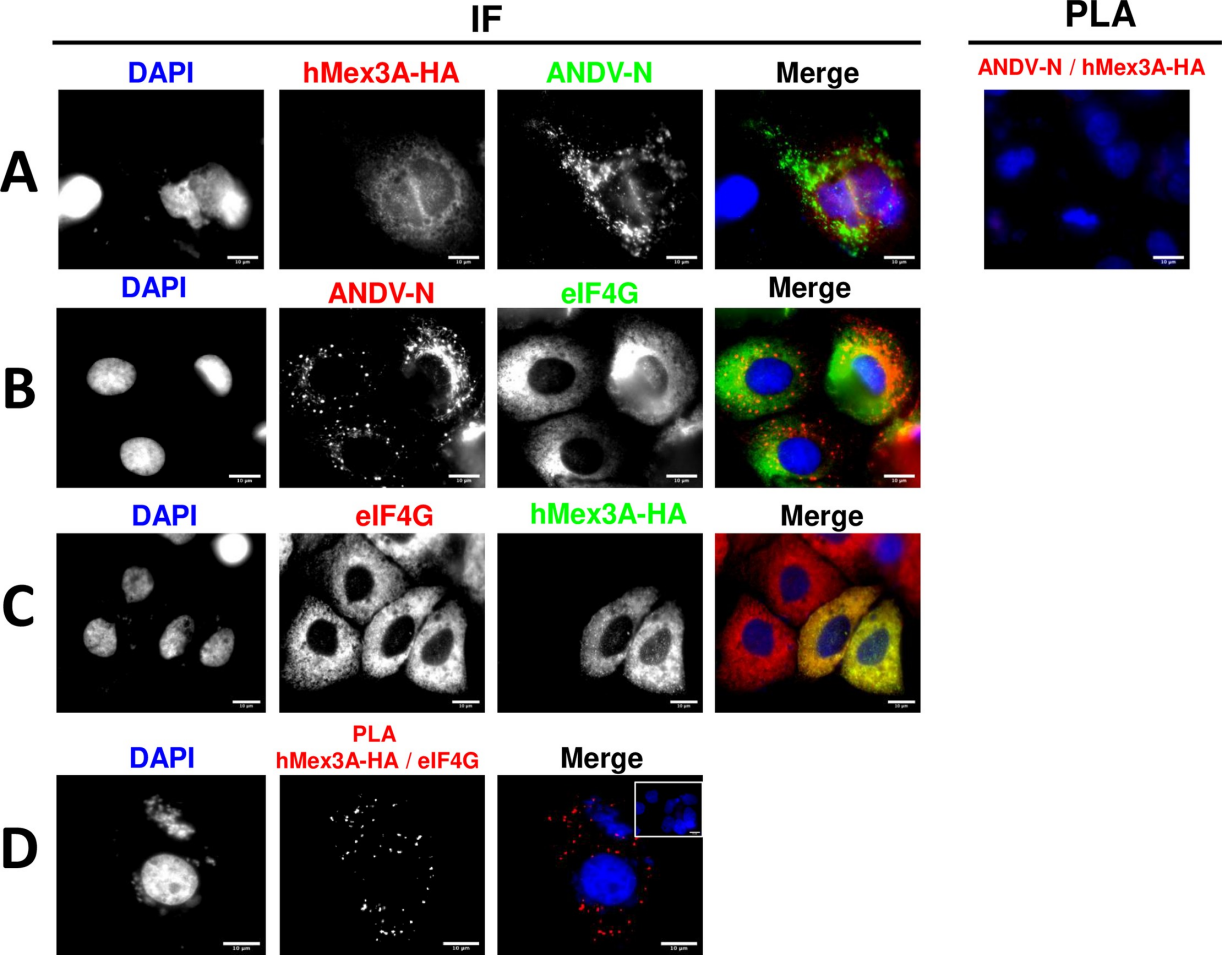
In ANDV-infected Huh-7 cells, hMex3A interacts with eIF4G, but not with the ANDV-N protein. **(A)** Huh-7 cells were infected with ANDV (MOI 1), and 24 hpi transfected with a plasmid encoding for hMex3A-HA protein. Twenty-four hpt cells were fixed (PFA 4%) and permeabilized (PBS-Triton). The ANDV-N and hMex3A-HA proteins were detected using a mouse monoclonal anti-ANDV-N or a rabbit polyclonal anti-HA as primary antibodies. For the IF, a donkey anti-mouse Alexa 488 or a donkey anti-rabbit Alexa 594 were used as the secondary antibody. For the hMex3A-HA/ANDV-N interaction assay, PLA secondary antibodies were used following the manufacturer’s instructions. **(B-D)** Huh-7 cells were infected with ANDV (MOI 1), and 24 hpi transfected with a plasmid encoding for hMex3A-HA protein. Twenty-four hpt cells were fixed (PFA 4%) and permeabilized (PBS-Triton). **(B)** The expression of the ANDV-N and eIF4G was confirmed by IF using a mouse monoclonal anti-ANDV-N and a polyclonal anti-eIF4G as primary antibodies and a donkey anti-mouse Alexa 594 and donkey anti-rabbit Alexa 488 as secondary antibodies, respectively. **(C)** Endogenous eIF4G and recombinant hMex3A-HA protein were detected by IF using a rabbit polyclonal anti-eIF4G antibody and a monoclonal mouse anti-HA antibody as primary, and an Alexa 594 donkey anti-rabbit or a donkey anti-mouse Alexa 488 as the secondary antibodies. As a negative control for the IF, the inset, in the upper right corner of the merge, shows ANDV-infected cells where detection was conducted using only the secondary antibodies. **(D)** ANDV-infected cells were incubated with primary antibodies against hMex3A-HA and eIF4G as in (C), but PLA secondary antibodies were used following the manufacturer’s instructions. In the upper right corner of the merge, the inset shows infected and transfected cells without primary antibodies but with the PLA secondary antibodies added as a negative control of PLA. **(A-D)** Vectashield with DAPI was used as mounting media. The images were obtained in Olympus epifluorescence Microscope and processed by Image J.

Next, we sought to establish if the ANDV-N and the hMex3A-HA protein interacted in ANDV-infected Huh-7 cells. For this, Huh-7 cells were infected with ANDV CHI-7913 (MOI of 1) and then transfected with a plasmid encoding an HA-tagged hMex3A protein. The expression of ANDV-N and hMex3A-HA proteins was confirmed by IF (Fig 9A). Unexpectedly, no PLA signal was detected when the ANDV-N and hMex3A-HA proteins were targeted (Fig 9A). This result indicated that even though present within the ANDV-infected cells, the ANDV-N and hMex3A-HA proteins do not directly interact. These findings also suggested that the impact of the ANDV-N and the hMex3A proteins on the rate of translation of the N-RNA-3’UTR RNA represented two independent, yet complimentary, events.

Knowing that eIF4G was required for SmRNA translation (Fig 2 and [27]) and that eIF4G favors canonical mRNAs 5’-3’end interaction [20,24,25], we wondered whether the hMex3A-HA protein could interact with eIF4G in ANDV-infected Huh-7 cells. Twenty-four hours post-infection of Huh-7 cells with ANDV CHI-7913 (MOI of 1), cells were transfected with the plasmid encoding for the hMex3A-HA protein. The expression of the ANDV-N (Fig 9B), the hMex3A-HA (Fig 9C), and the endogenous eIF4G proteins (Fig 9B and 9C) was confirmed by IF twenty-four hours post-transfection. The PLA results suggest that in ANDV-infected Huh-7 cells, the recombinant hMex3A-HA protein interacts with the endogenous eIF4G (Fig 9D).

**Fig 10:**
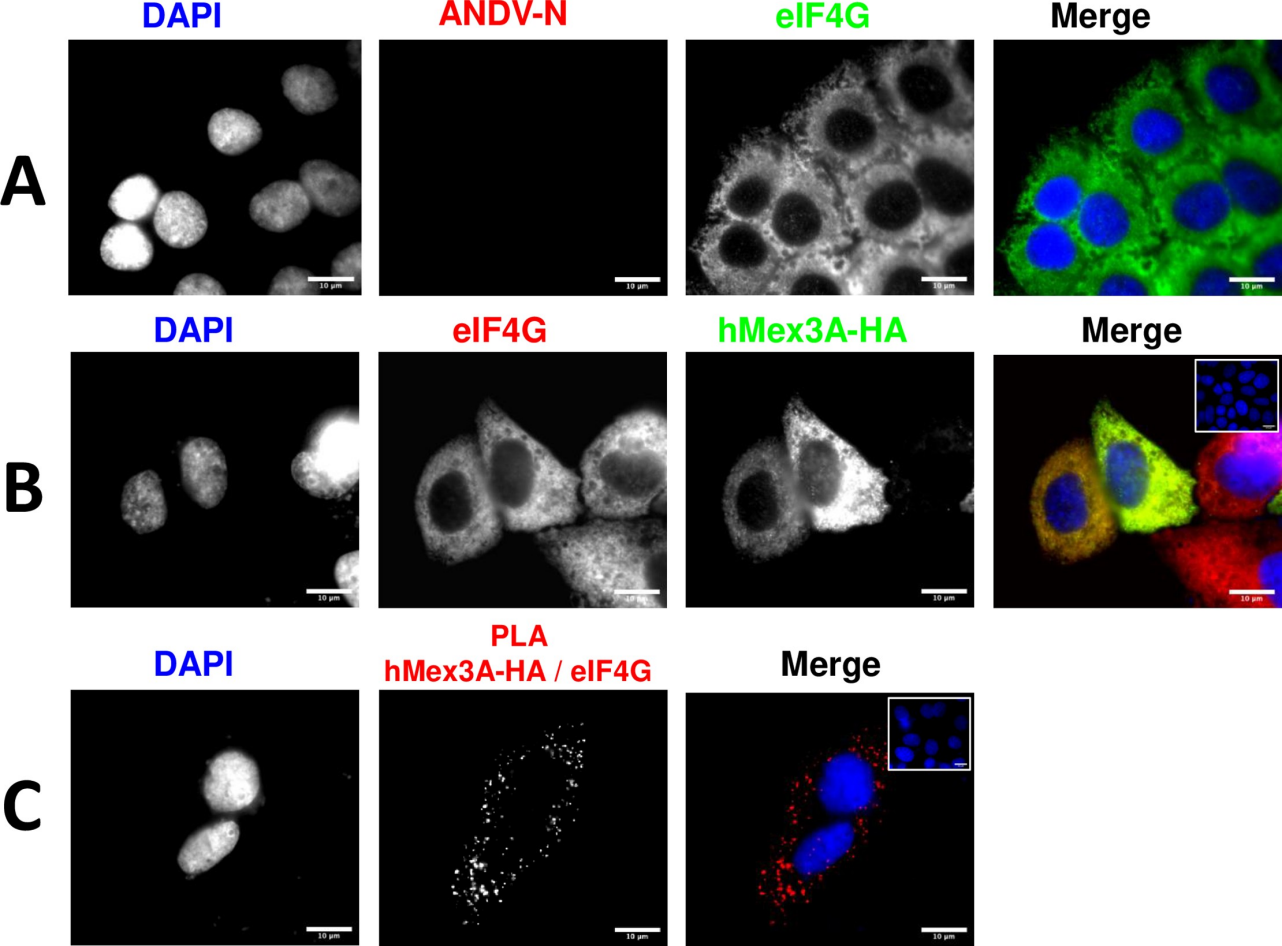
The hMex3A protein interacts with eIF4G in Huh-7 cells. Huh-7 cells were transfected with a plasmid encoding for the hMex3A-HA protein, and 24 hpt they were fixed using (PFA 4%) and permeabilized (PBS-Triton). **(A)** ANDV-N and the endogenous eIF4G proteins were detected by IF using a mouse monoclonal anti-ANDV-N or a rabbit polyclonal anti-eIF4G antibody as primary, and a donkey anti-mouse Alexa 594 or an Alexa 488 donkey anti-rabbit as the secondary antibodies. **(B)** The hMex3A-HA and the endogenous eIF4G proteins were detected by IF using mouse monoclonal anti-HA or a rabbit polyclonal anti-eIF4G as primary antibodies. As for secondary antibodies, a donkey anti-mouse Alexa 488 or a donkey anti-rabbit Alexa 594 were used. As a negative control of the IF, the inset in the upper right corner of the merge shows cells incubated only with secondary antibodies. **(C)** hMex3A-HA expressing Huh-7cells incubated with primary antibodies as in (B), but PLA secondary antibodies were used following the manufacturer’s instructions. As a negative control of the PLA, the inset in the upper right corner of the merge shows cells incubated only with the PLA secondary antibodies. **(A-C)** Vectashield with DAPI was used as mounting media. The images were obtained in Olympus epifluorescence Microscopy and processed by Image J.

Next, we wondered whether ANDV infection was a requirement for the hMex3A-eIF4G interaction in Huh-7 cells. For this, Huh-7 cells were transfected with the plasmid encoding for the hMex3A-HA protein. Twenty-four hours post-transfection, we confirmed that cells were not ANDV-infected by an IF against the ANDV-N protein using the detection of endogenous eIF4G as a control (Fig 10A). The expression of the hMex3A-HA was also evaluated by IF (Fig 10B). A PLA assay confirmed that the recombinant hMex3A-HA protein and the endogenous eIF4G are found in close proximity in non-infected Huh-7 cells (Fig 10C). Thus, results suggest that the hMex3A-eIF4G interaction was independent of ANDV infection.

**Fig 11:**
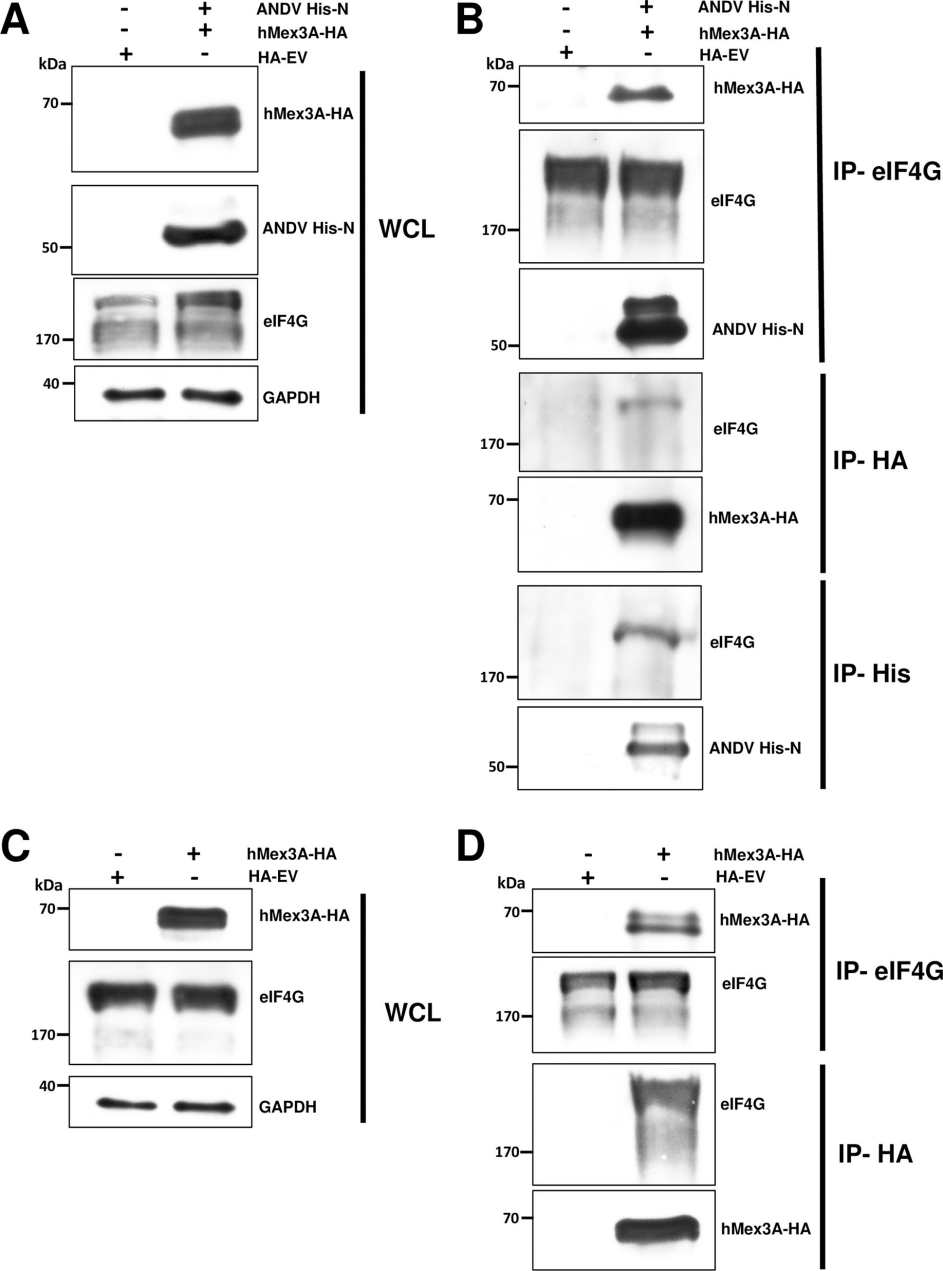
The hMex3A protein interacts with eIF4G in HEK 293T cells. HEK293T cells were cotransfected with the ANDV His-N and the hMex3A-HA **(A-B)**, or only with the hMex3A-HA **(C-D)** encoding plasmids. The HA-EV plasmid was used as a control **(A-D)**. Forty-eight hours later, cells were lysed, and immunoprecipitation (IP) assays were performed using protein A/G agarose coated with the corresponding antibody. The beads were washed extensively and incubated with loading buffer at 95°C, and the supernatant was used for western blotting. **(A-C)** Whole-cell lysate (WCL) of each sample and **(B-D)** IP fractions with the corresponding primary antibody highlighted on the right side of the panels. Western blotting was performed using mouse anti-HA, mouse anti-His, rabbit anti-eIF4G, and mouse anti-GAPDH. Horseradish Peroxidase (HRP)-conjugated Protein A/G was used to detect the primary antibodies.

To further validate our conclusions, we carried out reciprocal Co-IP experiments in HEK293T cells transfected with plasmids encoding for the ANDV His-N and hMex3A-HA or with the HA-EV. Forty-eight hours after transfection, cells were lysed, and the expression of hMex3A-HA, ANDV His-N, and endogenous eIF4G in the WCL was confirmed by western blotting using GAPDH as a loading control (Fig 11A). WCL were then subjected to Co-IP using anti-eIF4G, anti-HA, or anti-His antibodies. The anti-eIF4G antibody immunoprecipitated eIF4G, hMex3A-HA, and ANDV His-N (Fig 11B). The anti-HA antibody immunoprecipitated hMex3A-HA and eIF4G (Fig 11B). While the anti-His antibody immunoprecipitated ANDV His-N and eIF4G (Fig 11B). These results indicate that in HEK293T cells (Fig 11), as in Huh7 cells (Figs 9 and 10), eIF4G interacts with both hMex3A-HA and ANDV His-N, but hMex3A-HA and ANDV His-N do not interact. To further confirm these findings, HEK 293T cells were transfected with the hMex3A-HA encoding plasmid or with the HA-EV control. Forty-eight hours later, cells were lysed, and the expression of hMex3A-HA and the presence of the endogenous eIF4G in the WCL were confirmed by western blotting using GAPDH as a loading control (Fig 11C). WCL were then subjected to Co-IP using an anti-eIF4G or anti-HA antibody. Results confirm that in HEK293T cells eIF4G and hMex3A-HA interact (Fig 11D).

## Discussion

The N-protein of hantaviruses participates in several stages of the virus replication cycle, including viral mRNA translation [11,12]. Noteworthy, translation initiation from the ANDV SmRNA is dependent on the 5’cap structure and can occur in the absence of any viral protein (Figs 1–3, and [27]). Nonetheless, *in vitro*, the presence of the ANDV-N protein stimulated translation from the SmRNA in a cap-dependent fashion (Figs 1 and 2A). The ANDV-N protein’s ability to promote cap-dependent translation resembles features reported for the SNV-N protein [12], as it was not specific for the viral RNA (Fig 1). However, in contrast to the SNV-N protein, which can entirely replace the eIF4F complex, enabling translation even under conditions that suppress cap-dependent translation initiation [12], the ANDV-N protein was unable to restore the function of eIF4G *in vitro* (Fig 2B and 2C). Therefore, in our hands, the ANDV-N protein was incapable of replacing the eIF4F complex (Fig 2), showing that at least eIF4G is a cellular factor required for *in vitro* translation initiation of the viral SmRNA. So, in contrast to what has been reported for the SNV-N protein [12,55], *in vitro*, we see cap-dependent, eIF4G–dependent, ANDV-N protein-mediated enhancement of translation (Figs 1 and 2). Noteworthy, the capacity to stimulate translation is not restricted to the SNV-N or ANDV-N as the N-protein of other members of the *Bunyaviridae* family share a similar function [28]. The CCHFV N protein enhances mRNA translation in conjunction with the viral mRNA 5′ UTR [28]. However, unlike the SNV-N, but similar to the ANDV-N protein, the CCHFV-N requires eIF4G to stimulate translation [28]. However, we cannot discard the possibility that the differences between the ANDV-N and SNV-N proteins in their capacity to replace eIF4F are associated with the systems used to evaluate their functions. The SNV-N protein function was assessed in cells [12], while here, we assessed the capacity of the ANDV-N protein to replace eIF4G *in vitro*. Additionally, in our case, the recombinant tagged-ANDV-N protein was generated in bacteria and not in cells (Figs 1 and 2). Alternatively, we could conclude that even though SNV and ANDV are rodent-borne members of the *Hantaviridae* family of viruses, their N proteins act differently regarding their capacity to promote N-mediated translation. This conclusion would be consistent with data showing that the SNV-N and ANDV-N proteins are not structurally identical, and they do not share all biological functions [56–58]. The crystal structure differences between the core regions of the SNV-N and ANDV-N proteins suggest that the mobility of their N- and C- terminal bulge differs during the formation of high-ordered ribonucleoprotein complexes [56]. In terms of biological function, the ANDV-N protein inhibits interferon signaling response by regulating RIG-I, MDA5, MAVS, TBK1, and IKKε induced transcriptional responses from ISRE, κB, and IFN-β promoters, and also functions as an antagonist of Jak/STAT signaling, but the SNV-N protein does not [57,58].

In ANDV-infected cells, the ANDV-N interacted with eIF4G (Fig 4). The ANDV N-eIF4G interaction did not require any other viral components as it also occurred in virus-free cells overexpressing the ANDV-N protein (Fig 11). As *in vitro*, in cells, the ANDV-N protein stimulated translation from the viral-like SmRNA (Fig 3). However, the stimulation of capped-virus-like RNA translation mediated by the ANDV-N protein in cells depended on the presence of the 3’UTR of the ANDV SmRNA (Fig 3). Results also show that the poly(A) tail cannot fully recapitulate the 3’UTR function when the viral-like SmRNA is translated in the presence of the ANDV-N protein (Fig 3). Similarly, the CCHFV-N protein cannot stimulate translation from polyadenylated virus-like RNA [28]. A straightforward interpretation of these data is that the SmRNA of the *orthohantavirus* ANDV requires specific PABP-independent 5’-3’ end interaction for its optimal translation [27].

The ANDV is not the only RNA virus whose mRNA lacks a 3’poly(A) tail. More than one-third of positive-strand RNA plant viruses [59], the mRNA of some members of the *Flaviviridae*, and *Reoviridae*, and even the cellular mRNAs encoding for histones lack a 3’poly(A) tail [20,21]. Despite the apparent structural disadvantage, these viral and cellular mRNAs translate efficiently in cells. Interestingly, mRNAs lacking poly(A) tails adopt alternative poly(A)-independent strategies to establish contacts between their 5’ and 3’ ends [20,60–63]. Here we identified the hMex3A as a SmRNA 3’UTR interacting protein (Figs 5, 6 and 8, and S1 Data). Interestingly, when overexpressed alone, the hMex3A stimulates translation of the SmRNA in a 3’UTR dependent manner (Figs 7 and 8). Importantly, translation stimulation of the SmRNA-like RNA requires Mex3A to bind the viral RNA (Fig 8). The presence of several MREs in the 3’UTR of the SmRNA (Fig 6A) may account for this observation (Figs 7 and 8). Consistent with this possibility, no effect on translation was observed when the hMex3A G240D RNA-binding mutant was expressed (Fig 8). These observations highlight the role the 3’UTR plays in recruiting cellular proteins and the relevance of the 3’UTR for the adequate translation of the ANDV SmRNA. So data confirm that the SmRNA 3’UTR is more than a passive bystander in the process of viral mRNA translation [27].

In cells, hMex3A binds to the 3’UTR of its target mRNAs and localizes with them to P-bodies [44,50]. This is an exciting observation when analyzed in the context of *Hantaviridae* family replication. In P-bodies, the *orthohantavirus* N proteins bind 5′caps of cellular mRNA [5], and the viral RNA-dependent RNA polymerase snatches and uses the sequestered capped RNA fragments as primers to initiate transcription of the viral mRNA [5,64]. It is, therefore, plausible that during the step of viral RNA transcription, the ANDV-N and the hMex3A protein are independently loaded onto the SmRNA. As the ANDV-N protein and hMex3A do not interact (Figs 9–11), at some point between SmRNA synthesis and translation, both proteins have to be recruited to the SmRNA to enable the enhancement of viral protein synthesis (Figs 3, 7 and 8). Further experiments not included in this study are required to validate this hypothesis.

A totally unexpected finding of the study was the hMex3A-eIF4G interaction (Figs 9–11). The interaction was established in two different cell lines, Huh-7 (Figs 9 and 10) and HEK 293T (Fig 11), using different but complementary experimental approaches. The hMex3A-eIF4G interaction showed to occur in the absence of ANDV-N (Figs 10 and 11) or independently from the virus’s presence (Figs 9 and 10). The biological significance of this observation remains unclear. However, the hMex3A has a crucial role in tumorigenesis and malignant progression [65,66]. Maybe the hMex3A-eIF4G interaction is essential for hMex3A’s oncogenic potential. This hypothesis still has to be evaluated. However, in the context of ANDV infection, it is tempting to speculate that during viral replication, the SmRNA highjacks a pre-existing eIF4G-hMex3A complex to facilitate its translation. Further experiments not included in this study are needed to validate this possibility.

An issue that we did not directly evaluate in this study and could have helped establish a unique model to explain our results is the possibility that the ANDV-N protein could replace eIF4E as the cap-binding protein, as does the SNV-N protein [12,67]. So we can only conclude that both the 5’UTR and 3’ UTR participate in ANDV SmRNA translation, but we do not know how the SmRNA circularizes to favor translation. According to the closed-loop model, cellular mRNAs establish a 5’cap-eIF4F-PABP-3’poly(A) 5’-3’ interaction that stimulates translation synergically [22,68]. In the case of SmRNA-like RNAs harboring the 3’UTR, co-expression of the ANDV-N and the hMex3A additively stimulate translation from the virus-like mRNA in cells (Fig 8). Interestingly, the establishment of the cap-eIF4E-eIF4G-PABP-poly(A) interaction, expected for the capped virus-like mRNA harboring a poly(A) tail, would not enable ANDV-N (Fig 3) or hMex3A (Fig 7) stimulation of translation. So, data would suggest that the SmRNA 5’-3’ interaction alone or the formation of a circularized mRNA structure is insufficient for efficient ANDV-N and hMex3A mediated enhancement of SmRNA translation (Figs 3 and 7). Further studies are required to understand the molecular mechanisms behind these results.

Based on our findings alone, it is difficult to put forward a unique model of translation regulation that encompasses the viral SmRNA, eIF4G, hMex3A, and the ANDV-N protein. Also, we cannot discard the possibility that at least three independent and competing complexes are involved in translation of the SmRNA. One complex harboring the SmRNA, ANDV-N, and eIF4G, another containing the SmRNA, hMex3A, and eIF4G, and a third with SmRNA, ANDV-N, hMex3A, and eIF4G could be formed during ANDV replication. As the ANDV-N and the hMex3A proteins independently interact with eIF4G, this initiation factor is likely to be an essential mediator in the 5’-3’ end interaction during ANDV SmRNA translation (Fig 2 and [27]). Further experiments are required to validate this possibility fully. Another issue that remains to be clarified is the step(s) in translation that is being impacted by the SmRNA 5’-3’ end interaction, as either translation initiation, ribosome recycling, or both could be favored by the formation of the closed-loop [22].

In summary, in this study, we report that a 5’-3’ end interaction favors translation of the ANDV SmRNA. We also show that the ANDV-N protein and the cellular protein hMex3A independently interact with eIF4G, probably closing the SmRNA loop favoring viral protein synthesis. Thus, both viral and cellular proteins regulate ANDV gene expression by enhancing translation.

## Materials and method

### Cell culture and virus infections

HEK 293T, (ATCC, CRL-11268), HeLa (ATCC, CCL-2), LS174T (ATCC, CL-188), and Huh-7 (provided by R. Bartenschlager, University of Heidelberg, Heidelberg, Germany) cells were grown in Dulbecco modified Eagle’s medium (DMEM; #SH30022, HyClone, GE Healthcare Life Sciences, Logan, Utah, USA) containing 10% fetal bovine serum (#SH30910, Hyclone, GE Healthcare Life Sciences), 1% penicillin-streptomycin (1000 U/ mL) (#SV30010, Hyclone, GE Healthcare Life Sciences), and 1% amphotericin B (25 mg/ml) (#SV30078.01, Hyclone, GE Healthcare Life Sciences), at 37°C in a 5% CO_2_ atmosphere. For Huh-7 cells culture, 1% nonessential amino acids (Gibco BRL, Life Technologies Corporation, Carlsbad, CA, USA) was added. ANDV CHI-7913 isolate production and infection assays were performed at a multiplicity of infection (MOI) of 1, as previously described [69]. Viral replication was monitored by detecting the ANDV-N protein by immunofluorescence using monoclonal antibodies kindly provided by N. Tischler (Molecular Virology Laboratory, Fundación Ciencia & Vida, Santiago, Chile) as described in [10,69]. The work with ANDV was performed in a biosafety level 3 (BSL-3) facility at the Escuela de Medicina, Pontificia Universidad Católica de Chile.

### Plasmids

The N-DNA and N-DNA-3’UTR plasmids used to generate the mRNAs were previously described [10]. The control plasmid expressing *Renilla* luciferase (RLuc), as well as the plasmid used for the expression of the ANDV GST-N and ANDV His-N proteins, have been previously described [10]. To generate plasmid ANDV HA-N, the ANDV-N ORF was recovered from the plasmid ANDV Topo-His-N by PCR using forward JOS7 primer 5’-TACGTGAATTCATGAGCACCCTCCAAGAATTGC-3’ and reverse JOS8 primer 5’-TACGTGCGGCCGCCTACAACTTAAGTGGCTCTTGG-3’. The PCR product was digested with EcoRI and NotI restriction enzymes and cloned into pCI-Neo-HA. The pCI-Neo-HA and Glo-Fluc plasmid are described in [29,30,70] and were kindly provided by Ricardo Soto-Rifo (ICBM, Universidad de Chile). The 3BoxB plasmids were generously donated by M. Hentze (EMBL Heidelberg, Germany) [42]. The 3BoxB sequence was recovered by PCR using primers Forward-EcoRI (5’-GAATTCGGCCGCCTAAGT-3’) harboring an EcoRI restriction site and the Reverse-XbaI (5’-TCTAGATCGAGATAATATCCTCGATAGG-3’) bearing XbaI restriction site. The obtained PCR product was cloned in a pGEM-T Easy plasmid (Promega Corporation, Madison, WI, USA) generating the plasmid pGEM-T-3BoxB. Plasmid N-DNA-3UTR [10], was digested using EcoRI and XbaI, and used as the backbone to clone the 3BoxB fragment recovered by digestion of pGEM-T-3BoxB with EcoRI and XbaI, generating plasmid 5’UTR-3BoxB. The latter plasmid was digested with NdeI and EcoRI to release the 5’UTR, refilled using the Klenow fragment (Thermo Fisher Scientific Inc, Waltham, MA, USA), and ligated, generating plasmid 3BoxB. The 5’UTR-3BoxB plasmid was then digested with XbaI, and the SmRNA 3UTR flanked by XbaI restriction sites was inserted downstream of 3boxB to generate plasmid 5’UTR-3BoxB-3UTR. The plasmid 3BoxB was digested with XbaI, and the SmRNA 3’UTR was cloned downstream of 3BoxB, generating plasmid 3BoxB-3’UTR. The human Mex3A was amplified from genomic DNA of LS174T cells using the Pfu Turbo polymerase (Agilent Technologies, Santa Clara, CA, USA) and cloned into pcDNA3. First, exon 1 was amplified and ligated into the pBluescript vector with primers Forward 5’-AATCTCGAGGCTTTTGTTTCGCCATGCCTAG-3’ and Reverse 5’- AATGAATTCACCGGTGTCTTGATGTAGGTGTTGGTC-3’. Then, exon 2 was amplified with primers Forward 5’- AATACCGGTGAGGGGCGAGGAACCAGTGTTC-3’ and Reverse 5’- AATGTCGACGCATGGGGCACGGGGCTTAGG-3’ and ligated to exon 1, taking advantage of an endogenous AgeI site at the 5´ of exon 2. The hMex3A-HA expressing plasmid was generated by PCR, by adding a C-terminal HA tag as well as attB gateway adaptors with primers Forward 5’-AAAAAGCAGGC**T**CCACCATGCCTAGTCTAGTGGTATCTGG-3’ and Reverse 5’- AGAAAGCTGGGTTTAAGCGTAATCTGGAACATCGTATGGGTATGGGGAGAATATTCGGATGGCTTGCGTGG-3’. The PCR product was cloned into the pDONR221 vector by Gateway cloning following the manufacturer´s instructions (Life Technologies). Human Mex3A-HA was then recombined into a gateway compatible pcDNA3 vector. The final pcDNA3 hMex3A HA (hMex3A-HA) was used for experiments. In order to generate the G240D mutant of hMex3A, pDONR221 hMex3A-HA was amplified with primers Forward 5’-GGTGGGGCTGGTGGTGG**A**CCCCAAAGGGGCAACC-3’ and Reverse 5’-GGTTGCCCCTTTGGGG**T**CCACCACCAGCCCCACC-3’ using the Pfu Turbo Polymerase (Agilent Technologies), following the manufacturer´s instructions to introduce a G>A mutation in position 719 of the hMex3A ORF. The amplified vector was then purified and digested with restriction enzyme DpnI for 4 hours at 37°C, and transformed into competent bacteria. The resulting pDONR221 hMex3A G240D HA vector was recombined into a gateway compatible pcDNA3 vector. The final pcDNA3 hMex3A G240D HA was used for experiments. The sequence of all constructs used in this study was verified (Psomagen Inc., Rockville, MD, USA). WT and G240D *MEX3A* was PCR amplified (PFU Turbo Polymerase, Agilent Technologies) from pDONR221 versions (see above), introducing an upstream EcoRI site (Forward 5’-aaaGAATTCCCACCATGCCTAGTCTAGTGGTATCTGG-3’), and a downstream NotI restriction site removing the stop codon (Reverse 5’-aaaGCGGCCGCGGAGAATATTCGGATGGCTTGCGTGGC-3’). WT and G240D *MEX3A* were cloned via EcoRI/NotI into a pcDNA3.1 vector with a C-terminal 2X strep Flag tag. The resulting WT and G240D MEX3A-TAP constructs were PCR amplified (Forward 5’-AAAAAGCAGGCTCCACCATGCCTAGTCTAGTGGTATCTGG-3’; Reverse 5’-AGAAAGCTGGGTGTCATTTATCATCATCATCTTTATAATC-3’) and recombined into a pDONR221 vector (Gateway system, Invitrogen, Thermo Fisher Scientific Inc). Both hMex3A-TAP and hMex3A G240D-TAP were cloned into a lentiviral doxycycline-inducible vector (pTRIPz) via Gateway recombination. HEK 293T cells were transfected with third-generation lentiviral packaging plasmids pKGPIR, pVSVG, pRTR2, and either pTRIPz hMex3A-TAP or pTRIPz hMex3A G240D-TAP in a 1:1:3:5. Supernatants from HEK 293T cells expressing recombinant retroviral particles were collected 48 and 72 hours post-transfection, filtered (0.22 μm filter), and used to transduce LS174T cells. Positively transduced LS174T cells were selected using Puromycin (2 μg/mL; Thermo Fisher Scientific).

### Ribonucleoprotein complex immuno-precipitation (RIP)-RT-qPCR

RIP was performed following the protocol described in [53,54]. LS174T hMex3A-TAP or hMex3A G240D-TAP expressing cells were seeded, and after 24 hours, doxycycline (1 μg/mL) was added. After a 16 hour induction, cells were washed twice with ice-cold PBS, scraped in PBS, and centrifuged at 1200 rpm for 5 min at 4°C. Cells were then resuspended in one pellet volume of polysome lysis buffer (100 mM KCl, 5 mM MgCl2, 10 mM HEPES pH 7.0, 0.5% NP40, 1 mM DTT, 100 units/mL RNAse OUT, 400 μM VRC, Protease Inhibitors). Cells were lysed on ice for 5 min and then stored at -80°C for at least 1 hour. The lysate was then thawed and centrifuged at 13200 rpm for 15 minutes at 4°C. The supernatant was kept for the IP. Magnetic anti Flag M2 beads (Invitrogen), were washed 5X with NT2 buffer (50 mM Tris HCl pH 7.4, 150 mM NaCl, 1mM MgCl2, 0.05% NP40). Half of the lysate (normally around 150 μL) was supplemented with NT2 buffer (up to 1 mL) and with RNAse Inhibitors (200 units/mL RNAse OUT, 400 μM VRC, 1 mM DTT, and 20 mM EDTA). 50 μL of washed beads were added to each lysate, mixed briefly, and 100 μL of the mix was kept as 10% of input for each IP. The rest of the IP was left for 2 hours at RT with orbital shaking. After this incubation, the supernatant was discarded, and the beads were washed 4 times with ice-cold NT2 buffer. After the final wash was done, all liquid was removed from the beads, and 1 mL of TRIzol reagent (Invitrogen) was added to these as well as the inputs. RNA was extracted following the manufacturer’s instructions. RNA from hMex3A-TAP and hMex3A G240D-TAP RIP, as well as their matched inputs, was used for cDNA synthesis using the High Capacity cDNA Reverse Transcription Kit (Applied Biosystems, Thermo Fisher Scientific Inc) followed by a quantitative PCR (qPCR) using Power SYBR MasterMix (Applied Biosystems) targeting EED (forward: 5’-ATACGAGCCATCCTCTGCTG-3’; reverse: 5’-ATCTCTGTGCCCTTCTACGC-3’) and GAPDH (forward: 5’-GTGGTCTCCTCTGACTTCAACAG-3’; reverse:5’- GTCTCTCTCTTCCTCTTGTGCTCTT -3’) RNAs. Direct comparisons of the qPCR values between the RIP samples and their inputs were conducted. Differential RNA-binding between the hMex3A and its mutant was assessed for RIP samples compared to their input for hMex3A-TAP and hMex3A G240D-TAP.

### *In vitro* transcription and riboprobes

Capped monocistronic RNAs were directly transcribed using T7 RNA polymerase (mMESSAGE mMACHINE Kit, #AM1344, Ambion, Thermo Fisher Scientific Inc) from linearized plasmids. In brief, N-RNA-3’UTR ANDV was linearized with BamHI (Thermo Fisher Scientific Inc) and N-RNA plasmid was linearized with XbaI (Thermo Fisher Scientific Inc) [27]. The RNAs were synthesized by *in vitro* transcription conducted in a final volume of 20 μL according to the manufacturer’s instructions. The Acapped-RNA was synthesized using the mMESSAGE mMACHINE Kit (#AM1344, Ambion) but replacing the 2X T7 NTP/CAP mix for a homemade 2X T7 NTP/ACAP mix. The 2X T7 NTP/ACAP mix contained the A(5’)ppp(5’)G cap-analog (6 mM final concentration; #S1406S; New England Biolabs, Ipswich, MA, USA) and NTPs (7.5 mM final concentration of CTP, ATP, UTP and 1.5 mM final concentration of GTP; #R0471, #R0461, #R0451, #R0441, Thermo Fisher Scientific Inc). After two hours of synthesis, RNAs were treated with 1 μL of Turbo DNase (Ambion) for 30 min at 37°C. The poly(A) tailing kit (#AM1350, Ambion) was used to add poly(A) tail to the cap-N-RNA mRNA according to the manufacturer’s specifications. RNAs were precipitated for one hour with 2.5 M LiCl, centrifuged at 16 000 g for 30 min at 4°C, washed with 75% ethanol, resuspended in 25 μL of nuclease-free water, and purified through Sephadex G50 columns (Sigma Aldrich, Merck KGaA, Darmstadt, Germany). RNAs concentrations were determined spectrophotometrically (N60-Implen Nanophotometer, Implen GmbH, Munchen, Germany, USA), and RNA integrity was monitored by electrophoresis on denaturing agarose gels. For the generation of strand-specific riboprobes that target the ANDV SmRNA, first the ANDV-N coding region was amplified by RT-PCR with primers JOS63-Forward 5’-ATGAGCACCCTCCAAGAATTAC-3’ and JOS64-Reverse 5’- TTACAACTTAAGTGGCTCTTGG-3’ using total RNA extracted from ANDV CHI-7913 infected cells. The RT-PCR product was cloned in pGEM-T easy (Promega Corporation), and the orientation of the amplicon was confirmed by sequencing, making the plasmid pGEM-T-N-ANDV. The plasmid pGEMT-N-ANDV was linearized with NcoI (#ER0575, Thermo Scientific), for the transcription with SP6 RNA polymerase (#EP0131, Thermo Scientific), generating complementary riboprobes, which recognize the positive strand of ANDV SRNAs. One microgram of lineal plasmid was used for the *in vitro* transcription, following the manufacturer’s instructions and in the presence of 1X of DIG RNA labeling Mix (#11277073910, Roche Applied Science, Mannheim, Germany). After two hours of synthesis, RNAs were treated with 1 μL of Turbo DNase (Ambion) for 30 min at 37°C. RNAs were precipitated for one hour with 2.5 M LiCl, centrifuged at 16000 g for 30 min at 4°C, washed with 75% ethanol, and finally resuspended in 25 μL of nuclease-free water. All the RNAs were purified through Sephadex G50 columns (Sigma Aldrich), and RNAs concentrations were determined spectrophotometrically (N60-Implen Nanophotometer). The purified RNAs were fragmented using an RNA fragmentation kit (#AM8740, Ambion), using between 5 to 10 μg of total RNA resuspended in nuclease-free water to reach a volume of 30 μL and incubated with 3 μL of 10X Fragmentation buffer. The samples were incubated at 70°C for 15 min in a heating block, and then 3 μL of Stop solution was added to each tube and mixed carefully. The fragmented RNAs concentration was determined spectrophotometrically (N60-Implen Nanophotometer). Several aliquots of 25 ng/μL were made and stored at -80°C until use.

### *In vitro* translation

*In vitro* translation was carried out in nuclease-treated Flexi rabbit reticulocyte lysate (RRL) (Promega Corporation) at 35% vol/vol, supplemented with 20 μM amino acids (Promega Corporation) and 8 U/μL Ribolock RNase inhibitor (Fermentas, Thermo Fisher Scientific), with 80 mM of potassium acetate (KOAc), 0.5 mM of magnesium acetate (MgOAc_2_), different molar concentrations of ANDV GST-N protein (0.25:1 to 250:1 protein/RNA molar ratio) and mRNAs used at a final concentration in the reaction of 0.01 pmol. As a control, GST alone was added to the RRL in a 1:1 (protein /RNA molar ratio). Reactions were performed for 90 min at 30°C. For the foot-and-mouth disease virus (FMDV) L-protease assays, RNA of the FMDV L-protease was in vitro translated in nuclease-treated RRL as described above from the pLb plasmid (a kind gift from Graham Belsham, Department of Veterinary and Animal Sciences, University of Copenhagen, Denmark) [71]. In brief, 40 ng/μl of the FMDV L-protease uncapped RNA was translated in 50% (v/v) of RRL for 90 min at 30°C. The L-protease-RRL was diluted in fresh RRL 35% (v/v) in a ratio of 1:2 and 1:5. The diluted L-protease-RRL was added to fresh RRL used for the translation of the ANDV N-RNA to a final concentration of 4% (v/v) of Protease L [30,35–37]. Translation reactions were incubated at 30°C for 90 min. Luciferase activities FLuc and RLuc were measured using the DLR assay system (Promega Corporation) on a Sirius single-tube luminometer (Lumat 9507; Berthold Detection Systems GmbH, Pforzheim, Germany) as previously described [37]. Data are expressed as a percentage of the Relative Luciferase Activity (RLA) or as Relative Translation Activity (RTA); the latter corresponding to the FLuc/RLuc ratio.

A one-step RT-qPCR detected the ANDV N-RNA-3’UTR and Glo-Fluc-poly(A) mRNAs from translation reactions. Briefly, after *in vitro* translation, 1 μL of the final reaction was taken, and a 1/100 dilution in nuclease-free water was made. The RT-qPCR were carried out in an optical tube 8x strip (#401428; Agilent Technologies) using the Brilliant II SYBR Green qRT-PCR one Step Master Mix (#600835; Agilent Technologies), 1 μL of each 1/100 diluted mRNA and 200 nM of each primer in a final volume of 15 μl. The amplification program consists of 30 min of reverse transcription at 50°C, 10 min of initial denaturing at 95°C and followed by 35 cycles (20s denaturation step at 95°C, 20s of annealing at 60°C, and 30s of extension at 72°C) of complementary DNA cDNA amplification. FLuc RNA was detected with firefly sense (5’-ACTTCGAAATGTCCGTTCGG-3’) and firefly antisense (5’-GCAACTCCGATAAATAACGCG-3’) primers, as previously described (23,26,27). Ribosomal 18S rRNA, used as a reference gene, was detected with 18S sense (5’-GTGGAGCGATTTGTCTGGTT-3’) and 18S antisense (5’-CGCTGAGCCAGTCAGTGTAG-3’) primers. The 2^−ΔΔCt^ method was used for the relative quantification of RNA [72,73]. The reactions were carried out in a Stratagene Mx3000P thermal Cycler (Agilent Technologies), and the MXPro MX300P V4.10 Build 389, scheme 85 software was used to obtain the data.

### Recombinant protein expression

The GST-N protein or GST alone were expressed in the *Escherichia coli* BL21-CodonPlus (DE3)-RIPL bacterial strain (Stratagene, Merck KGaA). Bacteria expressing the recombinant proteins, which were previously induced with 1 mM isopropyl-β-D-thiogalactopyranoside (IPTG) for 6 h, were lysed on ice using lysis buffer (3 mg of lysozyme in 50mM Tris, pH 8.0, 200mM NaCl, 1mM EDTA, 5mM dithiothreitol [DTT], 1.5mM N-lauryl sarcosine, and protease inhibitors [Complete protease inhibitor cocktail tablets; Roche Applied Science]). Lysates were sonicated and clarified by centrifugation. The supernatant was combined with 1 ml of fresh glutathione-Sepharose 4B beads (GE Healthcare) and rotated at 4°C for 4h. The beads were then washed three times with washing buffer (50 mM Tris, pH8.0, 1mM EDTA, 1% Triton X-100, and 200mM NaCl). Elutions were obtained in 50mM Tris, pH8.0, 10mM glutathione. The eluted sample was next concentrated using Amicon Ultra-0.5 centrifugal filter device (Merck Millipore, Merk KGaA). The protein concentration and integrity were determined by a Bio-Rad protein assay (Bio-Rad Laboratories Inc., Hercules, CA) and SDS-PAGE, respectively.

The λ-GST protein was express as previously described [42]. Briefly, *Escherichia coli* BL21-CodonPlus (DE3)-RIPL bacterial strain (Stratagene) were transformed with the plasmid coding the λ-GST protein and induced with 1 mM isopropyl-β-D-thiogalactopyranoside (IPTG) for 2 hours at 37°C. Bacteria were then lysed on ice using lysis buffer (50mM Tris pH8.0, 500mM NaCl, 5% glycerol vol/vol and proteases inhibitors [Tablets; Roche Applied Science]). Lysates were sonicated (Sonic Ruptor 250 (Omni International, Kennesaw, GA, USA)) and clarified by centrifugation. The supernatant was incubated with glutathione-sepharose 4B beads (GE Healthcare) for 4 hours at 4°C with rotation. The beads were then washed three times with lysis buffer complemented with triton X-100 0.001% and DTT 1mM. Elution was performed using the same lysis buffer complemented with glutathione 20mM. The eluted sample was next concentrated using Amicon Ultra-0.5 10K centrifugal filter device (Merck Millipore), and the protein concentration and integrity were determined by a Bio-Rad protein assay (Bio-Rad Laboratories Inc., Hercules, CA), and SDS-PAGE and coomassie staining, respectively.

### GRNA affinity chromatography and NanoLC-MS/MS

These experiments were conducted according to the protocol described in [42]. Briefly, the RNAs containing the 3BoxB sequence by itself or flanked by either the ANDV SmRNA 5’UTR, ANDV SmRNA 3’UTR, or both sequences were incubated for one hour in HeLa cell extract at 37°C. The mix was then incubated for two hours in rotation with 150μL of glutathione beads bound to the recombinant λN-GST, and resuspended in GRNA buffer (20mM Tris pH 7.5, 150mM NaCl, 1.5mM MgCl_2_, 8.7% glycerol, 0.05% NP-40 and 12μg/mL heparin added fresh). Beads were washed three times with 500μL of GRNA buffer and incubated for 30 minutes at 37°C with 100 μg/mL of RNAse A. The supernatant was resolved in a gradient polyacrylamide gel to visualize the protein band pattern in each sample. The NanoLC-MS/MS was performed by Applied Biomics, Inc (https://www.appliedbiomics.com/) (Hayward, CA, USA) using the total amount of supernatant obtained in the GRNA affinity chromatography assay. For the sample preparation, proteins were exchanged into 50 mM ammonium bicarbonate buffer. DTT was added to a final concentration of 10mM and incubated at 60°C for 30 min, followed by cooling down to room temperature. Iodoacetamide was then added to a final concentration of 10mM and incubated in the dark for 30 min at room temperature. The proteins were then digested by Trypsin (Promega Corporation) overnight at 37°C. NanoLC was carried out using a Dionex Ultimate 3000 (Milford, MA). Tryptic peptides were loaded into a μ-Precolumn Cartridge and separated on an acetonitrile gradient (ranging from 5% to 60%) on a Nano LC column. Fractions were collected at 20-second intervals followed by Mass Spectrometry analysis on AB SCIEX TOF/TOF 5800 System (AB SCIEX, Framingham, MA, USA). Mass spectra were acquired in reflectron positive ion mode. TOF/TOF tandem MS fragmentation spectra were acquired for each ion, averaging 4000 laser shots per fragmentation spectrum (excluding trypsin autolytic peptides and other known background ions). For the Database search, both the resulting peptide mass and the associated fragmentation spectra were submitted to GPS Explorer workstation equipped with MASCOT search engine (Matrix Science, London, UK) to search the database of Swiss-Prot. Searches were performed without constraining protein molecular weight or isoelectric point, with variable carbamidomethylation of cysteine and oxidation of methionine residues, and with one missed cleavage also allowed in the search parameters.

### DNA and RNA transfections

HEK 293T cells (6 x10^4^ cells) were transfected with plasmids coding ANDV HA-N, ANDV His-N, hMEX3A-HA, and hMEX3A-HA G240D at 60% confluency using PEI (polyethyleneimine; GIBCO, Thermo Fisher Scientific Inc) in 48 well formats. 48 hpt, the cells were transfected with 0.15 pmol of capped-mRNA coding FLuc with 3’UTR or poly(A) tail using Lipofectamine 2000 (Thermo Fischer Scientific Inc). As a transfection normalizer, 0.15 pmol of the capped and polyadenylated *Renilla* Luciferase (RLuc) mRNA was cotransfected. Six hours post-RNA transfection, the cells were collected and lysed with passive buffer (Promega Corporation). The activity of FLuc and RLuc was measured in cell lysates (10 μL) using the DLR Assay System (Promega Corporation) according to the manufacturer’s instructions using a Sirius Single Tube Luminometer (Berthold Detection Systems GmbH). The remaining cellular lysates were used in western blotting assays. Experiments were conducted at least three times, each in triplicate. For immunoprecipitations experiments, 3.6 x10^6^ HEK 293T cells were seeded in 10 cm plate format and 24 hours later transfected with HA-EV or ANDV His-N and hMex3A-HA using 15 μg each vector by PEI. In co-immunoprecipitations experiments, plasmids ANDV His-N (7.5 μg) and hMex3A-HA (7.5 μg) were co-transfected in cells using PEI. 48 hours post-transfection cells were collected in lysis buffer (NaCl 100 mM, EDTA 2 mM, Tris-HCl 50mM pH 7.5, NaF 50mM, Sodium orthovanadate 1mM, Triton X-100 1%, Protease inhibitors (Roche Applied Science)). For RT-qPCR experiments from RNA transfected cells, 1.5 x10^5^ HEK 293T cells were seeded in 24 well plates, 24 hours later were transfected with DNA plasmids by PEI as above, and 48 hpt the N-RNA-3UTR or N-RNA-polyA RNAs were transfected using Lipofectamine. 6 hpt the cells were washed with PBS and lysed with 100 μL RLNa buffer (10mM Tris-HCl pH 8, 10mM NaCl, 3 mM MgCl_2_, 0,5% NP-40, 1 mM DTT) containing 10 U of Ribolock RNase inhibitor (#EO0381, Thermo-Fisher Scientific Inc.). After a 5 minute incubation on ice, 500 μL of TRIzol reagent (#15596018, Thermo Fisher Scientific Inc.) was added following the manufacturers’ instructions. Total RNA concentration was quantified by nano-spectrophotometry (N60-Implen Nanophotometer). 100 ng per sample was used for the RT-qPCR assay. The real-time RT-qPCR experiments were carried out using the Brilliant II SYBR Green RT-qPCR one Step Master Mix (#600835, Agilent Technologies). FLuc and GAPDH primer were used as previously described [27]. A qPCR with no previous RT step was carried out to control for contaminant DNA. Data analysis was performed by the previously described ΔΔCt method [72,73].

### Immunoprecipitations and Western blots

Protein concentration was determined by Bradford assay using the Bio-Rad protein assay (Bio-Rad Laboratories, Inc., Hercules, CA, USA). 30–60 μg of total protein was resolved on a 15% tricine–SDS-polyacrylamide gel and transferred to a polyvinylidene difluoride (PVDF) membrane (Hybond-P; GE Healthcare). The membrane was blocked with TBS-Tween 0,1% plus 5% skimmed milk for 1 hour and incubated overnight with primary antibodies at 4°C. As primary antibodies, an anti-polyhistidine monoclonal antibody (H1029; Sigma-Aldrich) or an anti-HA monoclonal antibody H9658 Sigma-Aldrich) and an anti-GAPDH antibody (#MAS-15738, Thermo Scientific) were used at a 1:5,000 dilution. An anti-mouse horseradish peroxidase (HRP)-conjugated antibody (074–1806; KPL Inc., Gaithersburg, MD) was used as a secondary antibody at a dilution of 1:10,000. For protein detection, the SuperSignal West Pico chemiluminescent kit (Thermo Fisher Scientific Inc) was used. For the L protease evaluation assay in Fig 1A, 10 μL of the final translation reaction was resolved in a gradient polyacrylamide gel. The gradient was generated between 6% and 15% concentration, using a light solution and a heavy solution (light solution: acrylamide/bisacrylamide 5%, 375 mM Tris-HCl pH 8.8, SDS 1%; heavy solution: acrylamide/bisacrylamide 15%, 375mM Tris-HCl pH 8.8, 275 mM sucrose), and then transferred to a (polyvinylidene difluoride) PDVF membrane (Hybond-P; GE Healthcare). The membrane was blocked with TBS complemented with Tween 0.05% and 5% skimmed milk overnight at 4°C. The membrane was incubated for 4 hours at 4°C with a mix of two primary antibodies targeting the anti-N terminal and anti-C terminal of eIF4G (kindly provided by Dr. Luis Carrasco, Centro de Biología Molecular Severo Ochoa, Madrid, Spain [74]) as in [30,35–37]. An anti-rabbit horseradish peroxidase (HRP) was used as a secondary antibody at a dilution of 1:10000 for 1 hour. For detection, membranes were incubated in a luminol:coumaric acid solution.

For co-immunoprecipitations experiments, total protein (1 mg) was used for immunoprecipitation assays together with protein A/G PLUS-Agarose (sc-2003, Santa Cruz Biotechnology, TX 75220, USA) previously loaded with anti-HA mouse monoclonal antibody (H9658, Sigma-Aldrich, St Louis, MO, USA), or anti-eIF4G rabbit polyclonal antibody (sc-11373, Santa Cruz Biotechnology, TX 75220, USA), or anti-polyhistidine monoclonal antibody (H1029; Sigma-Aldrich). For loadings of antibodies, protein A/G PLUS-Agarose was incubated with the antibody for 3 hours, and after the beads were washed once with lysis buffer to discard unbound antibody. Extracts were incubated with complex beads-antibodies for 16 hours at 4°C with rotation. The beads were washed with 1 mL of lysis buffer three times before directly mixing with tricine loading buffer (2X) and heated (95°C for 5min). The suspension was centrifuged, and the supernatant was recovered and subjected to western blot assay as described above but using protein A/G conjugated with HRP (Pierce # 32490, Thermo Fisher Scientific Inc) as the secondary antibody.

### Fluorescent in situ hybridization (FISH), immunofluorescence (IF) and proximity ligation assay (PLA)

For FISH-IF assay, cells were washed with PBS and fixed with 4% paraformaldehyde (PFA, Merck Millipore) in PBS for 10 minutes at room temperature. In the case of ANDV infected cells, after PBS wash, the cells were fixed with 4% PFA for 20 minutes at room temperature. Cells were permeabilized with 0.2% of Triton X-100 for 10 minutes at room temperature and washed with PBS once. Turbo DNAse (#AM2238, Thermo Fisher Scientific Inc) treatment was performed on the cover for 20minutes at 37°C. Covers were washed with PBS once and one wash with ultrapure water. Hybridization mix (50% deionized formamide (#F9037, Sigma-Aldrich), 1 μg/μL of *E*.*coli* tRNA, 2X SSPE buffer (#S2015, Sigma-Aldrich), 5X Denhardt’s solution (#D2532, Sigma-Aldrich), 2 mM vanadyl-ribonucleoside complex (VRC) (#94742, Sigma-Aldrich) and 50 ng of 11-digoxigenin-UTP probes (#3359247910, Roche Applied Science) in a humid chamber with 2X SSPE and 50% formamide (#K25168284, MERCK) overnight at 42°C in hybridization oven for 16hrs. Covers were washed with 50% deionized formamide-PBS for 30 minutes at 50°C and 20 minutes with 2X SSPE. Primary antibodies, namely, polyclonal sheep anti-Dig (#11333089001, Roche Applied Science) or monoclonal mouse anti-Dig (#11333062910, Roche Applied Science), mouse anti-HA (H9658; Sigma-Aldrich) or rabbit anti-HA (H6908; Sigma-Aldrich), rabbit anti-eIF4G (sc-11373, Santa Cruz Biotechnology), mouse anti-ANDV-N [69], in antibody dilution buffer (8% deionized formamide, 5X SSPE, 1X Denhardt’s solution, 2 mM VRC) were added for 1 hour at 37°C. Covers were washed twice with antibody dilution buffer for 10 minutes at 37°C. Alexa secondary antibodies donkey anti-sheep, anti-rabbit, anti-mouse were added for 1 hour at 37°C. Covers were washed once with FISH wash buffer (8% deionized formamide, 5X SSPE, and 2 mM VRC), two washes with PBS, and once with ultrapure water before adding Dako (#S3023, Dako North Amrerica Inc, CA, USA), without DAPI or Vectashield H1200 (Vector Laboratories, Inc, Burlingame, CA 94010, USA) with 4′,6-diamidino-2-phenylindole (DAPI) as the mounting medium, sealed with nail polish and stored at 4°C.

Immunofluorescence (IF) not involving RNA detection was conducted as in [69]. Briefly, covers were washed with PBS and fixed with 4% PFA for 10 minutes at room temperature. Coverslips were blocked with 10% BSA in PBS-Tr (PBS-Triton X-100 0.03%) for 1 hour at room temperature and incubated with primary antibodies in 5% BSA in PBS-Tr overnight at 4°C. Coverslips were washed 5 times with PBS-Tr and incubated with Alexa Fluor secondary antibodies at dilution 1: 250 (Invitrogen) for 2 hours at room temperature. Coverslips were washed 3 times with PBS-Tr, once with PBS, and once with ultrapure water. Vectashield with DAPI was used as a mounting medium. The Duolink *in situ* PLA kit was used (Duo 92004 and Duo 92002, Sigma-Aldrich) to determine the RNA-protein interactions in cells by RNA in situ hybridization coupled to PLA (RISH-PLA) [52]. Covers were incubated with primary antibodies as above and incubated with the PLA secondary antibodies anti-rabbit PLUS and anti-mouse MINUS for 1 hour at 37°C in antibody dilution buffer as above. Coverslips were washed 3 times with PBS and once with ultrapure water; the ligation (30 minutes at 37°C) and PCR assay (100 minutes at 37°C) were performed following the manufacturer’s instructions. Finally, coverslips were washed with PBS and ultrapure water before adding Vectashield H1200 with DAPI as a mounting medium. The PLA protocol, as described above, was used to determine protein-protein interactions in cells. Images were captured using an Olympus model BX51 epifluorescence microscope (60X objective) with MBF Stereo Investigator software version 11. ImageJ version 1.52c (National Institute of Health, USA) was used to split channels from raw images and to prepare images for publication.

## Supporting information

S1 Data Supplementary Dataset MS-Total. Datasets (DS) of proteins bound to (DS1) 5’UTR-3BoxB-3’UTR (pre-cleared with 3BoxB RNA), (DS2) 5’UTR-3BoxB (pre-cleared with 3BoxB RNA), (D3) 3BoxB-3’UTR (pre-cleared with 3BoxB RNA), (D4) 5’UTR-3BoxB-3’UTR (pre-cleared with 5’UTR-3BoxB RNA), shared (D1-D2), (D1-D3), (D1-D4), (D3-D4) proteins.
(XLSX)
